# Aptazyme-mediated gene regulation in *Strongyloides stercoralis* for functional studies of insulin receptor isoform specificity

**DOI:** 10.1371/journal.ppat.1013774

**Published:** 2025-12-17

**Authors:** Biying Zhang, Taoxun Zhou, Runxin Zhu, Peixi Qin, Juncheng Li, Chunqun Wang, Hui Liu, Min Hu

**Affiliations:** National Key Laboratory of Agricultural Microbiology, College of Veterinary Medicine, Huazhong Agricultural University, Hubei, P. R. China; University of Wisconsin-Madison, UNITED STATES OF AMERICA

## Abstract

Hammerhead ribozymes have found extensive applications in gene expression regulation across diverse biological systems including *Escherichia coli*, yeast, plants, and mammalian cells. However, their implementation in parasitic nematodes remains unexplored. *Strongyloides stercoralis* emerges as a particularly valuable model organism for studying developmental transitions in parasitic nematodes due to its unique life cycle alternating between parasitic and free-living stages. To expand the experimental toolkit for investigating developmental, evolutionary, and behavioral processes in this species, we established a conditional gene regulation system through transgenic integration of synthetic ribozyme constructs and demonstrated efficacy in regulating both exogenous (*mrfp*) and endogenous (*unc-22*) gene expression through targeted RNA processing mechanisms. Focusing on the insulin/IGF-1 signaling pathway, a critical regulator of parasitic nematode development and longevity, we implemented ribozyme-mediated post-transcriptional control to dissect functional divergence between two isoforms of the insulin receptor homolog *Ss*-DAF-2. Comparative analysis revealed isoform-specific characteristics: while both isoforms maintain conserved signaling functions, isoform B exhibits specific binding affinity for human insulin and demonstrates significant transcriptional upregulation during parasitic transition phases. This ligand selectivity profile suggests that isoform B may serve as a molecular interface for host-derived insulin signaling coordination during parasitism. This study established a programmable ribozyme tool in *S. stercoralis*, functionally discriminated the two *Ss*-DAF-2 isoforms through precision RNA engineering, and identified isoform-specific ligand preferences with implications for host-parasite signaling. Our findings not only validate ribozyme-based approaches for genetic manipulation in parasitic nematodes but also lay the groundwork for future implementation of synthetic RNA switches in helminth research.

## Author summary

Hammerhead ribozymes have been widely used as gene regulation tools in various biological systems, yet their potential in parasitic nematode research remains unexplored. In this study, we established the first synthetic ribozyme-based transgenic regulatory system in human and canine parasitic nematode, *Strongyloides stercoralis*. By targeting RNAs for degradation, we successfully regulated both exogenous (monomeric red fluorescent protein, *mrfp*) and endogenous (*Ss-unc-22*) gene expression. Focusing on the insulin/IGF-1 signaling pathway, which governs larval development and lifespan in nematodes, our analysis revealed functional differences between two isoforms of the insulin receptor homolog *Ss*-DAF-2. Notably, *Ss*-DAF-2B showed specific binding to human insulin and significant upregulation during developmental recovery following arrest. This ligand-selective feature highlights isoform B’s potential in orchestrating host-parasite interactions. Our work not only provides a new method for genetic manipulation in parasitic nematodes but also reveals the evolutionary strategy by which parasites hijack host insulin signaling to regulate their life cycles. The finding provides important clues for identifying novel anti-parasitic drug targets and marks a significant step forward in addressing antiparasitic drug resistance and advancing innovative therapeutic strategies against infections.

## 1. Introduction

Parasitic nematodes are widely distributed worldwide and represent a significant global health burden [1,2]. These pathogens infect plants, animals, and humans, severely threatening both public and veterinary health while causing substantial economic losses [2]. The intricate life cycle, substantial species diversity, and the increasing resistance to anthelmintic treatments underscore the critical need for novel control strategies that target the molecular regulators of nematode development and host-parasite interactions [3–5]. To achieve this goal, a deeper understanding of the biology of parasitic nematodes and the molecular mechanisms that control host invasion and developmental adaptation is essential.

Recent advances in artificial gene regulation systems provide promising research tools [6]. Ribozyme-mediated gene regulatory switches, particularly aptazymes combining hammerhead ribozymes with ligand-binding aptamers, have emerged as versatile genetic systems [7]. These synthetic RNA devices can precisely regulate gene expression through small molecule induction, overcoming the limitations of some gene editing systems that lack the ability to study molecular functions at specific growth phases [8–11]. Unlike natural ribozyme switches, aptazymes offer modular design capabilities with enhanced specificity and tunability [12,13]. It has been reported that most of the developed systems function as repressive switches, inhibiting gene expression upon ligand binding [14]. There are also some activation switches, but they are fewer in number and generally have weaker efficacy. Although they have been successfully applied to study the developmental processes and disease mechanisms in model organisms, the potential for studying host-parasite molecular interactions in parasitic helminths remains unexplored.

The clinically relevant nematode *Strongyloides stercoralis* presents distinctive advantages for studying parasitic adaptation. Its life cycle alternates between free-living and parasitic stages, with critical growth transition point influenced by both genetic factors and environmental cues [15,16]. The presence of free-living females enables application of transgenic techniques established in *Caenorhabditis elegans*, particularly microinjection-based transformation methods [17]. This unique biological feature positions *S. stercoralis* as an ideal model for studying molecular mechanisms underlying developmental transitions in parasitic nematodes [18,19].

In this study, we demonstrate the successful implementation of an artificial aptazyme system in *S. stercoralis*. Through gonadal microinjection of transgenic constructs containing fluorescent reporter and theophylline-dependent hammerhead aptazyme, we generated transgenic progeny capable of ligand-dependent gene regulation. Low-dose theophylline administration activated RNA cleavage activity, particularly downregulating target gene expression in larval stages. In addition, we found that this ribozyme could effectively manipulate the post-transcriptional regulation of isoforms in *S. stercoralis*. We demonstrate that the insulin receptor homolog *Ss-daf-2* encodes functionally distinct isoforms that play different roles in host-derived insulin signaling during parasitic transition. Our findings establish a powerful platform for the functional analysis of developmental-related genes. This breakthrough provides essential technical foundations for investigating critical gene functions during parasitic nematode development and host interaction processes.

## 2. Materials and methods

### 2.1 Ethics statement

According to the protocol (permit no. HZAUDO-2024–0004) approved by the Animal Ethics and Animal Experimentation Committee of Hubei Province, the *S. stercoralis* (UPD strain) was maintained in steroid-treated beagle dogs [20].

### 2.2 Genomic DNA and RNA extraction

Genomic DNA was isolated from 5,000–10,000 infective third-stage larvae (iL3s) using an EasyPure Genomic DNA kit (TransGen Biotech, Beijing, China). Freshly prepared DNA samples were either processed immediately or cryopreserved at -80 °C for subsequent applications [21]. Total RNA extraction was performed on ~ 10,000 iL3s using TRizol reagent (TransGen Biotech, Beijing, China) following standardized protocols. First-strand cDNA synthesis was then carried out with PrimeScript First-Strand cDNA Synthesis Kit (TransGen Biotech, Beijing, China) and stored at −80 °C until further analysis [22].

### 2.3 Transformation constructs and transformation of *S. stercoralis*

In this study, we employed the theophylline-regulated aptazyme system as the foundational platform for the ribozyme-aptamer complex. This system integrates the specific cleavage activity of ribozymes with the ligand-sensing capability of aptamers, enabling ligand-dependent regulation of the target RNA [8]. The designed complex contains two binding arms that are fully complementary to the selected target sequence, allowing precise binding to the RNA substrate via strict Watson–Crick base pairing [13,14]. The cleavage activity of the ribozyme depends on a conserved sequence requirement, namely the “NUX↓” motif, where N represents any nucleotide, U is uridine, and X can be any nucleotide except guanosine. Previous systematic studies have validated the influence of different nucleotide combinations flanking this cleavage motif on cleavage efficiency, providing an experimental basis for the rational selection of our target sites [13].

During the target sequence design process, multiple parameters were introduced to ensure effectiveness and specificity. First, stable RNA secondary structures were avoided in candidate sequences to prevent reduced accessibility due to folding. Second, the GC content of the binding arm region was controlled within the range of 40%–60% to balance binding affinity and sequence specificity. Finally, BLAST analysis was used to rigorously evaluate the homology of the selected sequences, ensuring high specificity toward the target gene and avoiding potential off-target effects or non-specific interference with other transcripts. 

Active or inactive variants of the theophylline-dependent aptazyme sequences were used to replace the GFP coding sequence in the original vector pAJ08 (*act2p::gfp*), pAJ20 (*rps21p::gfp*) and pPV230.13 (*era1p::gfp*), resulting in the recombinant plasmid pAJ08-HHR (Hammerhead ribozyme, HHR), pAJ20-HHR and pPV230.13-HHR. The original vectors pAJ08, pAJ20 and pPV230 were modified to carry plasmids encoding both green fluorescent protein (GFP) and red fluorescent protein (mRFP), resulting in recombinant plasmids pAJ09 (*act2p::gfp*, *act2p::mrfp*), pAJ21 (*act2p::gfp*, *rps21p::mrfp*), and pPV231 (*act2p::gfp*, *era1p::mrfp*) (S1A Fig). The HHR construct was diluted to 20 ng/μL, and the dual-fluorescence construct was also diluted to 20 ng/μL, then mixed for microinjection. The standard procedure for gonadal microinjection of constructs was carried out following the protocol in previous studies [23]. The transformed female and male worms were transferred onto a new NGM plate seeded with *E. coli* OP50. The transgenic first-stage larvae (F1) expressing mRFP were screened using a stereomicroscope (SZX16 Olympus). Worms were placed on a 2.0% agarose pad (Biosharp, Beijing, China) supplemented with 100 mM levamisole solution (Sigma, Aldrich) for immobilization. Worms imaged by Olympus BX53 microscope. Dual fluorescence was assessed at a laser-scanning confocal microscope (Nikon). Fluorescence images were converted to 8-bit grayscale in ImageJ (NIH, USA). A threshold was applied to generate binary images, and regions of interest (ROIs) were defined as specific organizations (body wall, intestine, or whole worm). The integrated fluorescence intensity of each ROI was then quantified and normalized to the corresponding area to account for size variation. Background signal was subtracted prior to quantification to reduce noise.

### 2.4 Quantitative real-time (RT) PCR analysis

RNA was extracted using a single worm RNA extraction method [24]. Briefly, 22 iL3s were transferred into 10 μL lysis buffer (5 mM Tris pH 8.0, 0.5% Triton X-100, 0.5% Tween 20, 0.25 mM EDTA and 1 mg/mL proteinase K) in 0.2 mL RNase-free PCR tubes. The tubes were incubated at 65 °C for 10 min, then 85 ℃ for 1 min, with immediate cooling on ice. The worm lysate was used for cDNA synthesis. cDNA was amplified using PrimeScript First-Strand cDNA Synthesis Kit (Takara, Tokyo, Japan). The expression profile of the target genes was detected on the CFX96 Touch Real-Time PCR Detection System (Bio-Rad, USA) according to the standard procedure. The reactions were programmed for 40 cycles at 95 °C for 5s and 60°C for 10s. The ΔCT approach was employed for quantitative comparison of gene expression, using *β-actin* as the endogenous control. The primer sequences for all the genes are provided in S1 Table.

### 2.5 Theophylline treatment

Theophylline (CAS 58-55-9, MACKLIN) was dissolved in sterile water (40 mM stock solution). Theophylline is directly added from the stock solution to liquid cultures. Worms carrying aptazyme-regulated reporters were cultured in the theophylline-containing liquid culture medium at indicated developmental stages at 20 ℃ for 24 h. To evaluate the applicability of theophylline as a specific ligand for genetic manipulation in *S. stercoralis*, we systematically assessed its toxic effects at various concentrations on both free-living adults (FL adults) and post free-living larvae (L1–L3). For the brood size assay, adult females were paired with males at a ratio of 1:2. Egg synchronization was not feasible in *S. stercoralis*. A mixed population of L1 and L2 larvae was used at 24 h and 48 h, whereas L3 larvae were examined at 72 h.

### 2.6 *Strongyloides stercoralis* larval motor ability assay

*Strongyloides stercoralis* F1 larvae were recovered from fecal-charcoal cultures using a Baermann apparatus [23]. Using an asteriomicroscope (SZX16 Olympus), the swimming of F1 in a dH_2_O drop was recorded for 10 s and the crawling on an NGM agar plate for 20 s [25,26]. The distance was calculated using the manual tracking plugin of Image J software, based on recording the frame-by-frame position changes of the marked worm’s centroid and calculating the total distance traveled. For the detection of twitching, the worms were placed in 10 μL of 1% levamisole hydrochloride solution diluted in dH_2_O [27,28]. After 3 min of treatment, the worms were observed under a dissection microscope for paralysis or twitching phenotypes. The percentage of twitching worms was calculated.

### 2.7 Sequence and phylogenetic analysis

We performed a Blast search on the National Center for Biotechnology Information (NCBI) (http://www.ncbi.nlm.nih.gov/BLAST/) and WormBase databases using GenBank accession number: *S. stercoralis* DAF-2 (AGC25444.1) sequences as queries and found sequences for *Drosophila melanogaster* (QQD79823.1), *Haemonchus contortus* (AID54910.1), *Caenorhabditis elegans* (NP_497650.4), *Parastrongyloides trichosuri* (ADN44512.1), Wormbase ID: *Necator americanus* (Necator_chrIII.pre1.g9420.t1), *Ancylostoma ceylanicum* (Acey_s0036.v2.g328.t1), *Strongyloides ratti* (SRAE_1000288200b.1), *Rhabditophanes sp.* (RSKR_0000957200.1). Sequence homology was assessed using the BLAST servers at the National Center for Biotechnology Information (NCBI) [29]. Evolutionary trees and bootstrap analysis were generated using the MEGA (Version 12). The sequences were aligned using the Clustal W program with default settings [30]. Phylogenetic analysis was performed by constructing a neighbor-joining (NJ) tree with bootstrap values greater than 70% reported [31]. Pairwise similarity of sequences was generated by TBTools (Version 2.360) based on protein sequences (Protein Pairwise Similarity Matrix). The HeatMap results were visualized using the HeatMap Illustrator-HeatMap tool in the Graphics module of TBTools [32].

### 2.8 Protein–protein docking

*Ss*-DAF-2b and *Hs*-INS were obtained from the Alphafold Protein Structure Database [33]. Molecular docking was performed using the HDOCK server, which utilizes a global search strategy based on fast fourier transform (FFT) to extensively explore potential binding modes of proteins. The server then refines all the collected binding modes through an iterative, knowledge-based scoring function [34]. The binding affinity and dissociation constants of the protein-peptide complexes were then predicted using the online server PRODIGY (https://wenmr.science.uu.nl/prodigy/) [35].

### 2.9 Yeast two-hybrid assay

For yeast two-hybrid assays, the coding regions of *Ss*-DAF-2A (1464 amino acid residues) and *Ss*-DAF-2B (1426 amino acid residues) were amplified from cDNA. The fragments were then subcloned into the plasmid pGBKT7 (Coolaber), which contains the GAL4 DNA-binding domain, resulting in the constructs pGBKT7-*Ss*-DAF-2A and pGBKT7-*Ss*-DAF-2B. *Hs*-INS sequence was directly synthesized and inserted into the pGADT7 vector, which contains the GAL4 activation domain, producing the plasmid pGADT7-*Hs*-INS. Yeast two-hybrid interaction assays were carried out following the manufacturer’s instructions (Coolaber). Competent cells (Coolaber) were cotransformed with either pGBKT7-*Ss*-DAF-2A or pGBKT7-*Ss*-DAF-2B along with pGADT7-*Hs*-INS. Yeast cells cotransformed with pGBKT7-Lam and pGADT7-T served as negative control, while those cotransformed with pGBKT7-P53 and pGADT7-T were used as positive control.

### 2.10 *Strongyloides sterocoralis* iL3 reactivation and feeding assay

Worms at the iL3 stage were isolated by the Baermann technique and washed twice with water and incubated with either M9 or 1640 buffer (RPMI; Gibco) [20,36]. For stimulation of feeding with insulin or HNMPA-(AM)_3_ treatment (human insulin, CAS 11061-68-0; bovine insulin, CAS 11070-73-8; porcine insulin, CAS 12584-58-6; HNMPA-(AM)_3_, CAS 120944-03-8; Aladdin), each condition used ∼300 iL3 in 1 mL in each well and incubated at 37 ℃ in 5% CO_2_ in air for 21 h. Then, 2 μL of FITC (CAS 3326-32-7; Solaibio) dissolved in N, N-dimethylformamide (DMF) (CAS 68-12-2; Sigma) was added at 20 mg/mL and incubated for 3 h [37]. After 24 h incubation, iL3 were collected and washed three times with M9 buffer. Only live iL3 with fluorescein isothiocyanate (FITC) in the pharynx were scored as “positive” for feeding. Three biological replicates were performed [38,39]. The human insulin was an animal-free recombinant product expressed in yeast. The bovine and porcine insulins were extracted and purified from the pancreatic tissues. Insulin or HNMPA-(AM)₃ was dissolved in DMSO and added to the culture medium at the appropriate concentration. The control group was treated with RPMI medium containing the corresponding concentration of DMSO, while the M9 buffer group was used as a negative control, representing “zero activation” with no nutritional or signaling stimuli.

### 2.11 Statistical analysis

Statistical analysis was performed using GraphPad Prism 9.0 software (GraphPad Software, USA), and standard deviation (s.d.) was calculated. One-way ANOVA was applied to compare the statistical differences between groups. Statistical probabilities of *p* < 0.05 were considered significant. A Mann-Whitney test or unpaired t-test with Welch’s correction was used for the group analysis. Statistical probabilities of *p* < 0.05 were considered significant. Two-way ANOVA analysis was used for multigroup analyses. Statistical probabilities of *p* < 0.05 were considered significant.

## 3. Results

### 3.1 The ribozyme-mediated cleavage induces the degradation of fluorescent reporter mRNA in *Strongyloides stercoralis*

**Fig 1:**
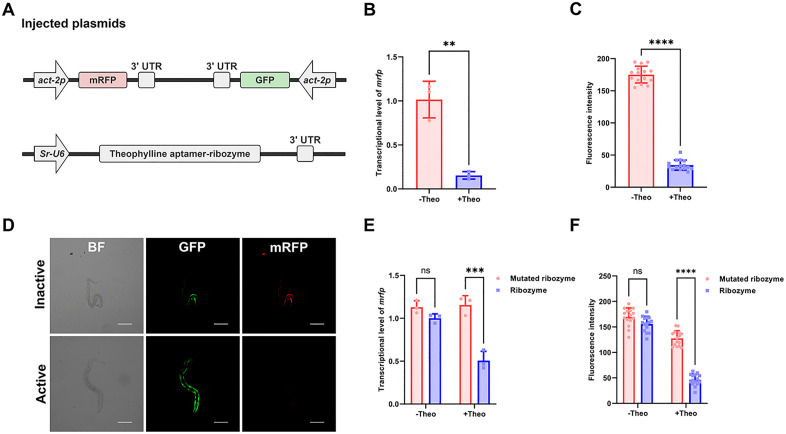
Theophylline-dependent ribozymes enable conditional gene expression in *Strongyloides stercoralis.* L2 larvae were treated with 1.5 mM theophylline for 24 h to induce ribozyme activity. (A) Schematic of the constructs microinjected into nematode gonads. The reporter vector includes a dual fluorescent reporters (*mrfp* as the target gene under ribozyme regulation and *gfp* as an internal control). The ribozyme vector contains a theophylline-responsive hammerhead ribozyme (HHR) module. (B) Relative mRNA abundance of *mrfp* under ribozyme switch regulation measured by quantitative real-time (RT) PCR. 22 worms per group were used and experiments were carried out in biological triplicates. Statistical significance was determined using the unpaired two-tailed t-test. Error bars, s.d. **p < 0.01. (C) Fluorescent intensity of reporter (mRFP) activity was quantified based on fluorescent imaging. n = 15. Statistical significance was determined using the unpaired two-tailed t-test. Error bars, s.d. **p < 0.01, ****p < 0.0001. (D) Microscope images of transgenic worms carrying ribozyme-regulated mRFP reporter. Scale bar = 100 µm. (E) Relative mRNA abundance of *mrfp* in catalytic core mutants measured by quantitative real-time (RT) PCR. 22 worms per group were used and experiments were carried out in biological triplicates. Statistical significance was determined using the two-way ANOVA, ns = not significant, Error bars, s.d. ***p < 0.001. (F) Fluorescent intensity of reporter (mRFP) activity in catalytic core mutants was quantified based on fluorescent imaging. n = 15. Statistical significance was determined using the two-way ANOVA, ns = not significant, Error bars, s.d. ****p < 0.0001.

To investigate whether ribozymes can exert regulatory functions in *S. stercoralis*, we selected a Ribo-Off tool validated and suitable for use in eukaryotic cells (S1B Fig). The ligand-dependent cleavage activity of the artificial hammerhead ribozyme (HHR) is a core design feature that enables its function as a molecular “switch”. The canonical natural hammerhead ribozyme is composed of three stem-loop regions (I, II, and III), where stems I and II form the catalytic core [40,41]. A stable aptamer structure, selected in vitro under high Mg²⁺ conditions, was integrated into stem-loop III. This integration spatially isolates the catalytic core, thereby effectively suppressing the ribozyme’s spontaneous cleavage activity [42]. When the ligand specifically binds to the aptamer domain, it induces a significant conformational rearrangement. This rearrangement physically disrupts the base pairs that stabilize the inactive state, thereby releasing the catalytic core and promoting its folding into the correct, active three-dimensional structure, which subsequently activates the ribozyme’s catalytic function. This hammerhead ribozyme (HHR) functions by inducing cleavage activity through the theophylline ligand [43,44]. Theophylline, a widely used small-molecule drug with low toxicity in eukaryotes and cost-effectiveness, was employed as the inducer. *Strongyloides stercoralis* worms exhibited robust tolerance to theophylline at concentrations below 1.5 mM (S2 Fig). To evaluate ribozyme cleavage activity on gene expression, we constructed a transgenic vector, fused with a 107 bp theophylline aptamer-ribozyme sequence. Using microinjection technology, the vectors containing artificial aptamer-ribozyme element and dual fluorescent reporters encoding mRFP (as the reporter) and GFP (as the reference) (Fig 1A), were delivered into the gonads of free-living female to generate transgenic progeny. As expected, upon addition of theophylline to the culture medium, the ribozyme in larvae carrying the HHR construct was activated, specifically cleaving the target mRNA (Fig 1B), leading to a significant reduction in mRFP transcription levels and consequently a decrease in protein expression (Fig 1C). Concurrently, strong GFP fluorescence was observed in these larvae (Fig 1D). Moreover, we mutated the active site of the ribozyme. It was found that, compared to the active ribozyme, worms with the inactive ribozyme in the presence of theophylline showed no significant change in fluorescence (Fig 1E). The ribozyme lost its cleavage activity and could no longer be regulated by theophylline to control the expression of mRFP gene (Fig 1F). These findings demonstrate that this ribozyme switch shows robust specificity and effective regulation in *S. stercoralis*.

### 3.2 The ribozyme switch enables temporal and spatial control of gene expression in *Strongyloides stercoralis*

**Fig 2:**
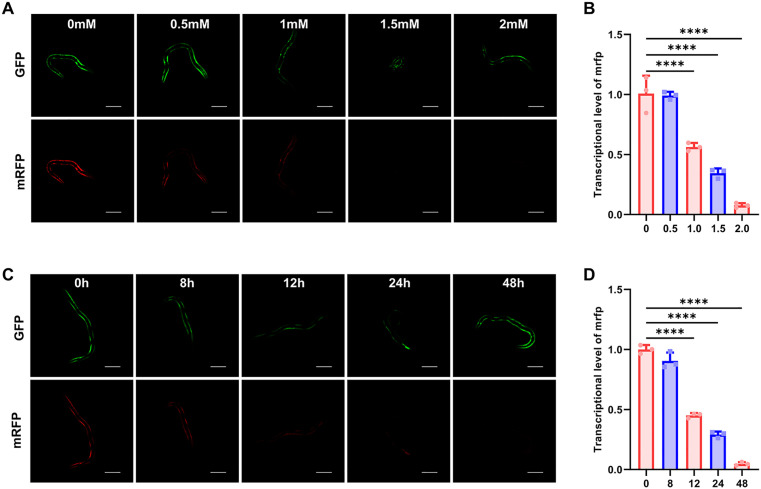
Spatial and temporal regulation of target gene transcription can be attained by ribozyme switch. (A) Confocal microscope images show ligand concentration-dependent regulation of *mrfp* transcription by ribozyme switch. Scale bar = 100 µm. (B) Quantitative real-time (RT) PCR analysis of *mrfp* transcription level under different concentrations of theophylline treatment. 22 worms per group were used and experiments were carried out in biological triplicates. Statistical significance was determined using the one-way ANOVA. Error bars, s.d. ****p < 0.0001. (C) Time-dependent effect of theophylline on gene expression regulation. Confocal images of larvae treated with theophylline for different time points. Scale bar = 100 µm. (D) Quantitative real-time (RT) PCR of *mrfp* in larvae treated with theophylline for different time points. 22 worms per group were used and experiments were carried out in biological triplicates. Statistical significance was determined using the one-way ANOVA. Error bars, s.d. ****p < 0.0001.

The regulatory effect of the ribozyme switch on the target gene is impressive. To further validate whether the ribozyme switch can function stably in *S. stercoralis*, we also tested the reaction activity of the ribozyme switch with the ligand. The second-stage larvae (L2) harboring active HHR-mRFP constructs were exposed to increasing theophylline concentrations. After 24 h, the mRFP fluorescence intensity decreased dose-dependently (Fig 2A-2B). At 1 mM theophylline, the ribozyme switch exhibited significant activation, suppressing mRFP fluorescence intensity to 69.6. While 2 mM theophylline achieved its peak (fluorescence virtually undetectable), although this concentration induced observable larval toxicity (S3 Fig). The regulatory ability of the ribozyme switch was tested at different time points with 1 mM theophylline. The results revealed progressive suppression of reporter gene expression, with enhanced regulatory fold-changes over time (Fig 2C). The effect of the ligand on the aptamer ribozyme was already very significant at 24 h and 48 h. After the addition of theophylline, mRFP fluorescence intensity was inhibited to 57.5 and 29.2, respectively (S3 Fig). Relative quantitative RT-PCR confirmed rapid theophylline-induced downregulation of mRFP transcripts (Fig 2D). These data confirm concentration- and time-dependent ribozyme activation in *S. stercoralis*. By utilizing the ligand to regulate its cleavage activity, conditional gene expression can be achieved.

**Fig 3:**
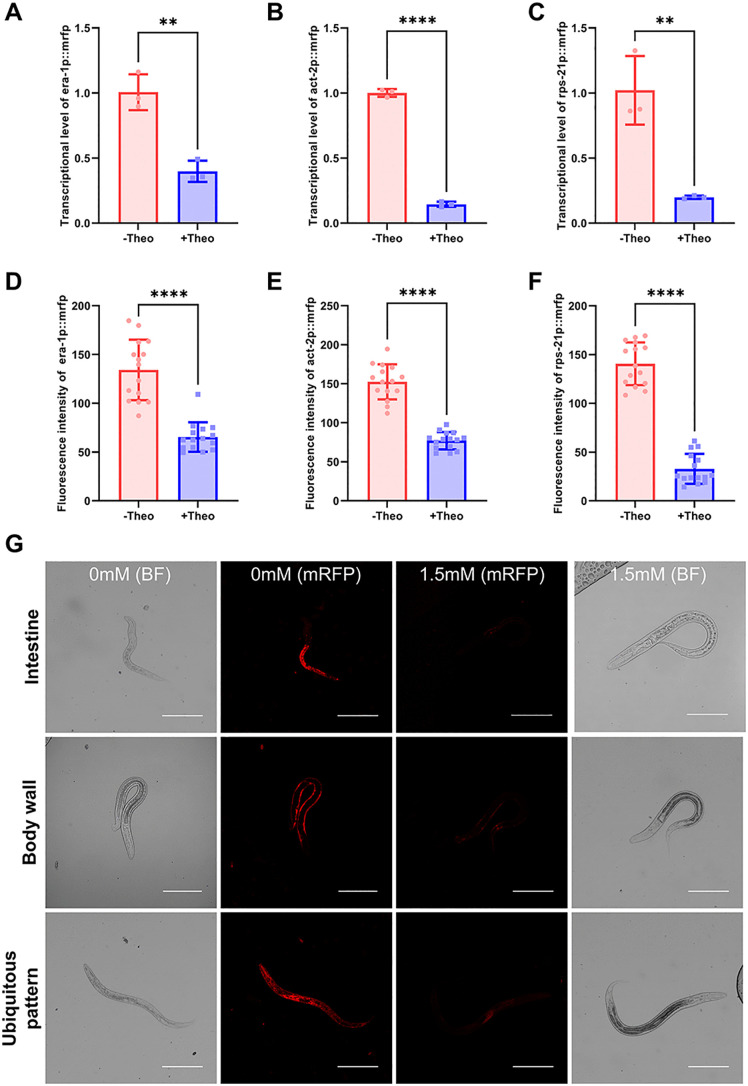
The artificial aptamer ribozyme is applicable for controlling transgene expression in various tissues of *Stongyloides stercoralis.* L2 larvae were treated with 1.5 mM theophylline for 24 h to induce ribozyme activity. Quantitative real-time (RT) PCR analysis of ribozyme-mediated *mrfp* repression under (A) intestine promoter (*era-1p*), (B) body wall muscle promoter (*act-2p*), and (C) general somatic cell promoter (*rps-21p*), respectively. Larvae were treated with 1.5 mM theophylline for 24 h. Three biological replicates were quantified and shown. Quantification of fluorescence intensity of ribozyme regulated *mrfp* expression driven by (D) intestine promoter (*era-1p*), (E) body wall muscle promoter (*act-2p*), and (F) general somatic cell promoter (*rps-21p*). n = 15. Statistical significance was determined using the unpaired two-tailed t-test. Error bars, s.d. *p < 0.05, **p < 0.01, ***p < 0.001, ****p < 0.0001. (G) Microscope images of worms carrying ribozyme-regulated mRFP reporters driven by tissue-specific promoters. Scale bar = 100 µm.

To evaluate spatial regulation, tissue-specific promoter *era-1p* (intestine), *act-2p* (body wall muscle), and *rps-21p* (general somatic cell) were used to drive aptazyme-regulated mRFP expression (Fig 3A-3E). Upon theophylline treatment, the aptamer-ribozyme effectively modulated target gene expression in tissue-specific cells. At the 24 h, the degree of mRFP inhibition between the intestinal wall and body wall localized ribozymes was comparable (50.6% vs. 48.7%), indicating that ribozymes function with similar efficiency in different tissue. This broad applicability suggests that ligand uptake and distribution may not impose strong tissue-specific constraints under these conditions (Fig 3). These data demonstrate that the ribozyme switch has good tissue generalization in *S. stercoralis*, supporting its utility for spatial regulation of gene expression in various tissues of the nematode.

### 3.3 Introduction of non-coding RNA scaffold stabilizes ribozyme switch structure in *Strongyloides stercoralis*

**Fig 4:**
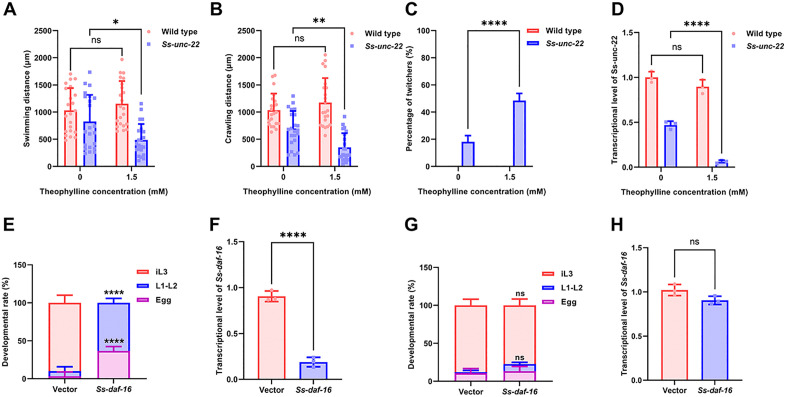
The ribozyme successfully suppressed gene expression in a controlled manner in *Strongyloides stercoralis.* L2 larvae were treated with 1.5 mM theophylline for 24 h. (A) Swimming distance of wild-type iL3s vs *unc-22* F1 iL3s over a 10 s period. (B) Crawling distance of wild-type iL3s vs *unc-22* F1 iL3s over a 20 s period. (C) The twitching phenotype in *S. stercoralis*. F1 was observed in 1% levamisole hydrochloride solution diluted in dH_2_O. n = 22 for each group and experiments were carried out in biological triplicates. (D) Quantitative real-time (RT) PCR confirmed ribozyme-mediated regulation of *Ss-unc-22* transcription. Three biological replicates were quantified and shown. Statistical significance was determined using the two-way ANOVA. Error bars, s.d. ns = not significant, *p < 0.05, **p < 0.01, ****p < 0.0001. (E) Developmental progression statistics of transgenic F1 larvae at various stages. n = 50 for each group and experiments were carried out in biological triplicates. Statistical significance was determined using the two-way ANOVA. Error bars, s.d. ****p < 0.0001. (F) Relative mRNA abundance of *Ss-daf-16* measured by quantitative real-time (RT) PCR. Three biological replicates were quantified and shown. Statistical significance was determined using the unpaired two-tailed t-test. Error bars, s.d. ****p < 0.0001. (G) Developmental analysis of transgenic F1 larvae following RNA scaffold-mediated stabilization of the ribozyme. n = 50 for each group and experiments were carried out in biological triplicates. Statistical significance was determined using the two-way ANOVA. Error bars, s.d. ns = not significant. (H) Transcript-level validation of *Ss-daf-16* by the stabilized ribozyme. Statistical significance was determined using the unpaired two-tailed t-test. Error bars, s.d. ns = not significant.

To validate that the conditional expression system can regulate endogenous genes in *S. stercoralis*, we targeted *Ss-unc-22*. The *unc-22* gene in nematodes encodes twitchin, a sarcomere-associated protein essential for stabilizing thick filaments during muscle contraction. Loss-of-function mutations result in uncoordinated locomotion (“twitcher” phenotype), characterized by erratic body bends and impaired movement due to destabilized myofilament lattices [27]. By integrating the ribozyme with the low-toxicity small molecule theophylline, we achieved *cis*-regulation of *unc-22* mRNA through modulated ribozyme cleavage. The selected transgenic offspring were cultured on solid and liquid media to observe their motility. The study found that both media exhibited motor dysfunction, with swimming and crawling speeds significantly lower compared to the wild-type strain (Fig 4A-4B). Relative quantitative RT-PCR confirmed ribozyme-mediated regulation led to a decrease in *Ss-unc-22* transcription (Fig 4D). Next, when exposed to acetylcholine receptor agonists, the larvae showed a twitching phenotype similar to that seen in *Ce-unc-22* mutants of *C. elegans* (Fig 4C). These results demonstrate that the ribozyme switch can modulate endogenous gene expression in *S. stercoralis*, establishing a tunable, low-toxicity gene regulation tool. Notably, even in the absence of theophylline (0 mM), target gene expression was markedly reduced. This suggests that the inactive conformation of the ribozyme was not fully stabilized intracellularly, leading to a baseline level of ‘leaky’ self-cleavage, which in turn caused the degradation of the mRNA (Fig 4D). This suggests that we should evaluate the unintended impact of ribozyme self-cleavage on the experiment.

To test whether ribozyme would interfere with the study of essential endogenous gene functions in the absence of ligand molecules, we targeted a key developmentally regulatory gene *Ss-daf-16*. The *daf-16* gene encodes a FOXO-family transcription factor that acts as the central effector of the insulin and insulin-like growth factor 1 (IGF1) signaling (IIS) pathway [45]. We fused a sequence targeting *Ss-daf-16* in the ribozyme switch (S1B Fig) and microinjected it into the gonads of free-living females to generate transgenic offspring. The transgenic progeny targeting *Ss-daf-16* failed hatching in most eggs (36.7%). Even when larvae hatched, they only developed to the L2 before dying (63.3%), and no larvae progressed to iL3 (Fig 4E). The results of relative quantitative RT-PCR indicate that ribozyme mediated self-cleavage affects the basal expression of target gene (Fig 4F). These results indicate that when ribozyme regulates important genes related to the development of *S. stercoralis*, its instability prevents larvae from reaching the developmental stage required for studying gene function. To address this, we introduced a human U15 non-coding RNA scaffold to enhance ribozyme structural stability. As expected, the stabilized ribozyme didn’t alter the basal transcription levels of target genes in ligand-free conditions (Fig 4G-4H).

### 3.4 Human insulin promotes the recovery of iL3 by activating insulin receptor *Ss*-DAF-2

**Fig 5:**
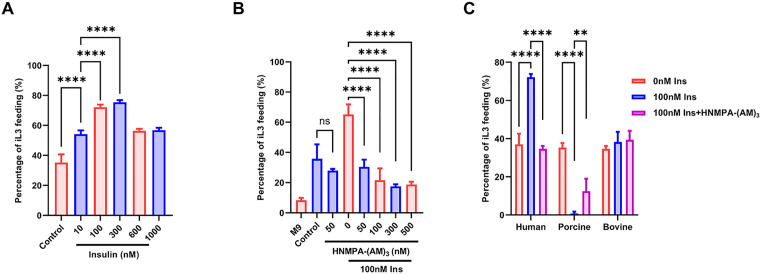
Human insulin promotes recovery of the dauer-like iL3 stage in *Strongyloides stercoralis*, this effect is suppressed by insulin receptor inhibitor treatment. (A) Infective third-stage larvae (iL3s) were cultured in RPMI-1640 medium supplemented with human insulin. Feeding recovery was assessed by monitoring the ingestion of FITC into the pharynx. DMSO was used as control. (B) Larvae were co-treated with human insulin and increasing concentrations of the insulin receptor inhibitor HNMPA-(AM)₃, to determine whether receptor blockade weakened the insulin-induced activation. Negative control larvae were treated with M9 buffer. DMSO was used in control. (C) Human insulin was compared with porcine and bovine insulin under equivalent concentrations in RPMI-1640 medium, to assess species-specific effects. n = 35-45 for each group and experiments were carried out in biological triplicates. Statistical significance was determined using the one-way ANOVA and two-way ANOVA. Error bars, s.d. **p < 0.01, ****p < 0.0001.

This ligand-inducible expression system allows for temporal-specific control of gene expression. Consequently, it serves as a powerful tool for studying molecular mechanisms underlying recovery from developmental arrest in parasitic nematodes during invasion of its definitive host. Previous studies have shown that the insulin signaling pathway is crucial for the development of iL3 in *S. stercoralis* during infection [45]. Since resumption of feeding in iL3 of *S. stercoralis* is a marker of further development [37], we supplemented nematode culture media with human insulin to investigate host-parasite molecular interactions by monitoring iL3 feeding reactivation. Gradual increases in insulin concentration (0–300 nM) revealed a dose-dependent enhancement of feeding resumption. At 10 nM, the activation rate was 54.2%, while at 300 nM, the activation rate significantly increased to 75.4%, indicating a positive correlation between insulin concentration and feeding recovery. However, when the insulin concentration exceeded 300 nM, feeding recovery plateaued, declining to levels equivalent to 10 nM treatment. Under 600 nM and 1000 nM conditions, the activation rates were 56.4% and 56.8%, indicating a non-monotonic dose-response relationship (Fig 5A). This phenomenon may imply either receptor saturation or activation of inhibitory pathways that counterbalance insulin’s stimulatory effects. In follow-up experiments, co-treatment with insulin receptor inhibitor, HNMPA-(AM)_3_, which completely eliminated the insulin-induced feeding reactivation, demonstrated that insulin’s effect is mediated through specific receptor activation. To assess whether HNMPA-(AM)₃ influences activation independently of exogenous insulin, we treated control iL3s with HNMPA-(AM)₃ in the absence of insulin stimulation. HNMPA-(AM)₃ is an insulin receptor inhibitor and the culture medium may provide a weak basal activation of insulin signaling, we compared feeding resumption between treated and untreated groups. The results showed that HNMPA-(AM)₃ treatment caused a slight reduction in the activation rate of iL3s; however, this difference was not statistically significant (*p* = 0.6) (Fig 5B). These findings confirm that human insulin promotes iL3 feeding resumption via activation of *Ss*-DAF-2, the important receptor in the IIS pathway. We hypothesize that *Ss*-DAF-2 plays a critical role in the mechanism of feeding recovery in iL3.

To determine whether *Ss*-DAF-2 exhibits ligand specificity for host-derived insulin or broadly responds to mammalian insulins, we tested porcine insulin (differing from human insulin by a single C-terminal residue) and bovine insulin (differing by three amino acids) for their effects on iL3 activation. Only human insulin significantly enhanced the feeding rate of iL3 (72.2%), showing a distinct species-specific response. Interestingly, porcine insulin markedly suppressed larval feeding activation (0.7%), while bovine insulin exhibited no significant effect (38.2%) (Fig 5C). The ligand discrimination shows the unique structural features of the interaction between human insulin and *Ss*-DAF-2, which appears evolutionarily tuned to selectively recognize human insulin as a host-specific developmental cue.

**Fig 6:**
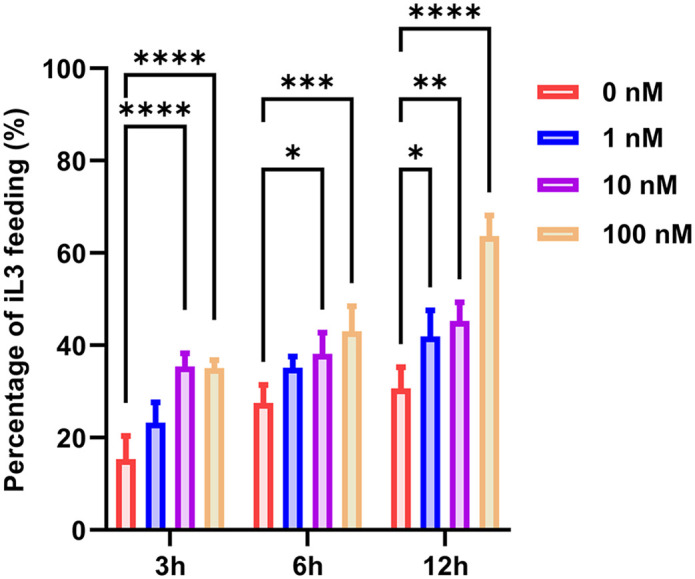
Dose and time dependent effects of human insulin on the resumption of feeding in infective third-stage larvae (iL3s). iL3s were incubated with human insulin at concentrations of 1 nM, 10 nM, and 100 nM for 3, 6, or 12 h, and the proportion of larvae resuming feeding was quantified. Human insulin stimulated iL3 activation in both a concentration- and time-dependent manner. Significant feeding resumption was observed at 10 nM and 100 nM insulin as early as 3 h, while 1 nM insulin promoted feeding only after 12 h of treatment. n = 27-34 for each group and experiments were carried out in biological triplicates. Statistical significance was determined using the two-way ANOVA. Error bars, s.d. *p < 0.05, **p < 0.01, ***p < 0.001****p < 0.0001.

We systematically evaluated the temporal and dose-dependent effects of human insulin on the resumption of feeding behavior in infective larvae (iL3s). iL3s were exposed to human insulin at concentrations of 1 nM, 10 nM, and 100 nM, and the proportion of larvae that resumed feeding was assessed at 3, 6, and 12 h post-treatment. The results showed that human insulin-mediated promotion of iL3 activation increased with both longer exposure time and higher concentration, showing a clear dose- and time-dependent response. Under short-term exposure, the high-dose groups exhibited the most significant response. Both 10 nM and 100 nM human insulin significantly stimulated iL3s to resume feeding within 3 h, with a statistically significant difference compared to the control group (****p < 0.0001). This indicates that higher concentrations of insulin can rapidly initiate signaling pathways, breaking the metabolic arrest in iL3s and inducing a transition to feeding behavior. In contrast, the 1 nM group did not show a significant increase in feeding at either the 3 h or 6 h. However, with extended incubation time, the effect of low-dose insulin became apparent. After 12 h of treatment, even 1 nM insulin significantly promoted the resumption of feeding in iL3s, demonstrating a clear difference compared to the control (*p < 0.05) (Fig 6). This result suggests that at physiological concentrations, the insulin signal requires a longer duration to accumulate, yet it still possesses sufficient biological potency to activate iL3s.

### 3.5 Human insulin enhances iL3 recovery through activation of insulin receptor *Ss*-DAF-2B

**Fig 7:**
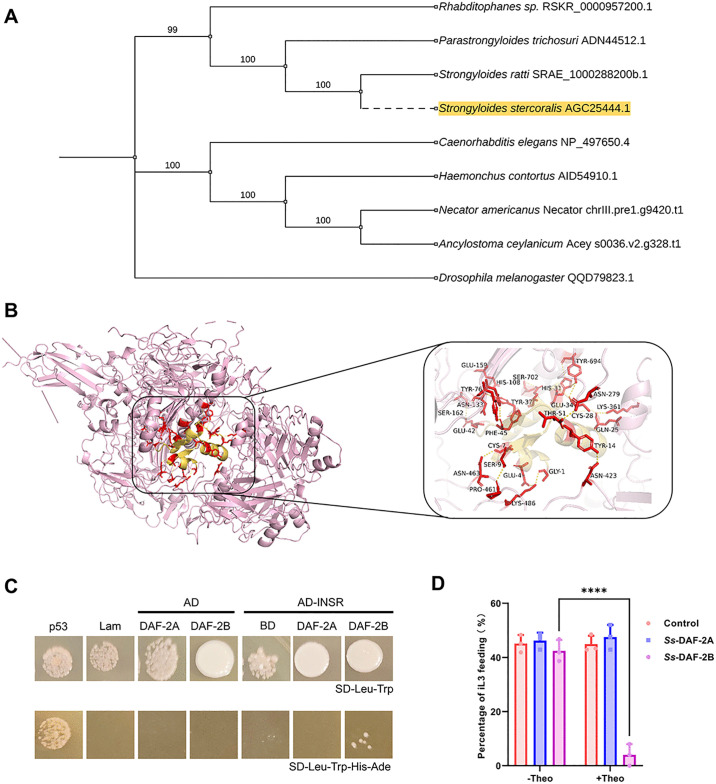
Insulin signaling plays a crucial role in regulating iL3 development by selectively activating *Ss*-DAF-2B. (A) To examine the phylogenetic relationship between insulin-like receptor ligand-binding domain of *S. stercoralis* and that of other species, a phylogenetic tree was constructed using the neighbor-joining method. Bootstrap analysis was performed with 1,000 replicates, and node support values are displayed a range from 0 to 1. The tree was generated by MEGA (Version 12) software analysis using Clustal W alignment with manual editing. (B) Molecular docking modeling between *Ss*-DAF-2B and *Hs*-INS using HDOCK. Pink indicates the *Ss*-DAF-2B protein, and yellow indicates the *Hs*-INS protein. (C) Results of yeast two-hybrid experiments. Translational fusions were generated for the Gal4 activation domain (Gal4-AD) and the human insulin *Hs*-INS. The Gal4 DNA binding domain (Gal4-BD) was fused to the insulin receptor *Ss*-DAF-2A and *Ss*-DAF-2B. Yeast strains were double transformed with the plasmid constructs and growth under different stringency conditions was assessed. (D) iL3s were treated with 1.5 mM theophylline together with 10 nM human insulin in the culture medium, and assessed activation after 12 h of treatment. The activation of iL3 was determined by the ingestion of FITC into the pharynx. n = 25-35 for each group and experiments were carried out in biological triplicates. Statistical significance was determined using the unpaired two-tailed t-test. Error bars, s.d. ***p < 0.001.

Previous studies have shown that *Ss-daf-2* has two selective splice variants [46]. We investigated whether *Ss*-DAF-2A and *Ss*-DAF-2B exhibit different ligand binding patterns, potentially forming a unique mechanism for their rapid adaptation to the distinct environmental cues of free-living and parasitic lifestyles. A neighbor-joining phylogenetic analysis of insulin-like receptor ligand-binding domains (LBDs) revealed distinct evolutionary relationships among *S. stercoralis* and related species. The patristic distance from *S. stercoralis* to both *A. ceylanicum* and *N. americanus* is comparable and longer than the distance from *C. elegans* to *S. stercoralis* (Fig 7A). Sequence alignment of the LBD revealed variations in amino acid identity across species (S4 Fig). The identity between *S. stercoralis* and hookworms was 56.24% for *A. ceylanicum* and 56.98% for *N. americanus*, while the identity with *C. elegans* was 51.09%. Within Clade IV, the identity between *S. stercoralis* and its congener *S. ratti* was very high (98.51%), whereas it was considerably lower with the free-living relative *R. diutinus* (52.91%) [47].

Both *Ss*-DAF-2A and *Ss*-DAF-2B share the same LBD in the extracellular region, but differ in their proteolytic processing sites. *Ss*-DAF-2A retains the canonical RIKR furin cleavage site, while *Ss*-DAF-2B disrupts this motif by retaining exon 2, introducing two alternative basic motifs (KNKK and RNKK). These differences may influence their roles in regulating the dauer stage and host-parasite interactions [48]. To evaluate the interaction between human insulin (Ins) and the *Ss*-DAF-2B receptor protein, we performed molecular docking analysis using HDOCK. As shown in Fig 7B, Ins formed a stable complex with *Ss*-DAF-2B. We focused on the interactions between amino acid residues such as ASN279, ASN463 and PRO461, which are primarily mediated by salt bridges and hydrogen bonds to enhance structural stability. Thermodynamic analysis revealed the binding free energy (ΔG) of the Ins-DAF-2B complex is -33.4 kcal/mol, with a dissociation constant (Kd) of 3.1 × 10 ⁻ ²⁵ M, indicative of high affinity and robust binding stability. In addition, the amino acid residue contacts within the complex were performed, with particular attention to the number of contacts between charged polar residues and nonpolar residues. The contact numbers for the Ins-DAF-2B complex were 58 and 102, respectively. These data underscore the decisive roles of both electrostatic and hydrophobic forces in maintaining the structural stability of the complex. Yeast two-hybrid assays confirmed direct interaction between human insulin and *Ss*-DAF-2B, but not *Ss*-DAF-2A (Fig 7C). We also used a yeast two-hybrid assay to detect the interaction between the two isoforms of *Ss*-DAF-2 and the endogenous insulin peptides of *S. stercoralis* (S5 Fig). *Ss*-DAF-2A displayed broad binding capacity, showing specific interactions with *Ss*-ILP-1, *Ss*-ILP-3, *Ss*-ILP-4, and *Ss*-ILP-7. In contrast, the *Ss*-DAF-2B isoform exhibited selective binding to *Ss*-ILP-6. The isoform-specific ligand selectivity may suggest evolutionary functional specialization allowing parasitic nematodes to distinguish self-generated developmental signals from host-derived activation cues, ensuring stage-specific developmental transitions.

We then performed in vivo functional validation using the ribozyme to regulate the endogenous gene expression in *S. stercoralis*. Since the *Ss-daf-2* gene has two isoforms (*Ss-daf-2a* and *Ss-daf-2b*) at the transcriptional level, which differ only in one exon, we designed the hybridization arms of the ribozyme based on this specific region, enabling precise binding and selective targeting of the mRNA of a specific isoform. Using an engineered theophylline-inducible aptazyme system, we achieved temporally dependent transcriptional regulation of *Ss-daf-2a* and *Ss-daf-2b*, respectively. This system allows for ligand-dependent intervention of gene expression at different developmental stages of the parasite, thereby providing a tool for us to investigate the role of insulin signaling in the recovery process of iL3s. We treated iL3 with theophylline together with 10 nM human insulin in the culture medium, and assessed activation after 12 h of treatment. The experimental results showed that under culture conditions supplemented with human insulin, larvae with normal expression of *Ss-daf-2b* were able to resume feeding, whereas when the expression of *Ss-daf-2b* was downregulated, the proportion of iL3s recovering feeding was significantly reduced. In contrast, downregulation of *Ss-daf-2a* under the same conditions did not lead to a significant defect in feeding recovery (Fig 7D). Collectively, these data demonstrate that human insulin promotes iL3 developmental progression through selective activation of the insulin receptor isoform *Ss*-DAF-2B.

### 3.6 Human insulin-mediated regulation of *Ss-daf-2b* expression enhances the sensitivity to future influx in *Strongyloides stercoralis*

**Fig 8:**
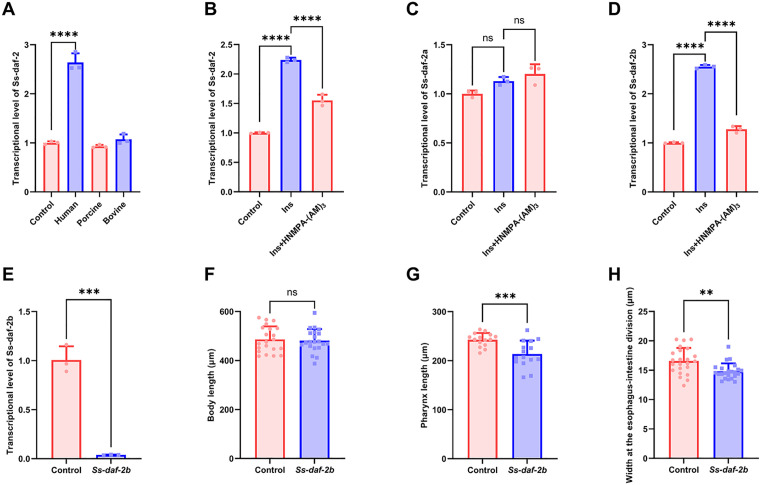
Human insulin regulates *Ss-daf-2b* transcription. (A) Relative mRNA abundance of *Ss-daf-2* under the treatment of insulins from different species. Relative mRNA abundance of (B) *Ss-daf-2,* (C) *Ss-daf-2a,* (D) *Ss-daf-2b* treatment with human insulin alone and in combination with the insulin receptor inhibitor HNMPA-(AM)₃. Three biological replicates were quantified and shown. Statistical significance was determined using the one-way ANOVA. Error bars, s.d. ns = not significant, ****p < 0.0001. (E) Relative quantitative RT-PCR confirmed ribozyme-mediated regulation of *Ss-daf-2b* transcription. Three biological replicates were quantified and shown. Statistical significance was determined using the unpaired two-tailed t-test. Error bars, s.d. ***p < 0.001. (F) Body length (n = 21), (G) pharynx length (n = 15), and (H) body width (n = 23) were measured using an Olympus SZX16 stereo microscope equipped with integrated cellSens standard measurement tools. Statistical significance was determined using the unpaired two-tailed t-test. Error bars, s.d. ns = not significant, **p < 0.01, ***p < 0.001.

Based on the finding that the host insulin activates *Ss*-DAF-2B to drive iL3 developmental progression, we next investigated whether receptor-mediated signaling modulates the expression of the insulin receptor (InR) through a feedback pathway, as observed in the conserved nutrient sensing pathway [49,50]. First, we examined the transcriptional impact of insulins from different species on *Ss-daf-2*. Human insulin significantly upregulated *Ss-daf-2* transcript levels (a 2.6 fold increase vs control), while porcine and bovine insulins showed no significant effects (0.9 and 1.0 fold changes, respectively) (Fig 8A), demonstrating species specificity in insulin recognition by *Ss-*DAF-2. We further studied isoform-specific transcriptional responses, revealing that human insulin elevated total *Ss-daf-2* transcription (Fig 8B) and human insulin selectively enhanced *Ss-daf-2b* transcripts, whereas *Ss-daf-2a* remained unaffected (Fig 8C-8D). Co-treatment with the insulin receptor inhibitor HNMPA-(AM)_3_ abolished this isoform-specific upregulation, confirming receptor-dependent transcriptional amplification.

To study the functional role of *Ss-daf-2b* in the developmental stages, we employed ribozyme-mediated regulation of *Ss-daf-2b* expression. Relative quantitative RT-PCR showed that activation of the ribozyme switches effectively induced downregulation of the target gene *Ss-daf-2b* (Fig 8E). Despite suppressed *Ss-daf-2b* expression, larvae progressed to the iL3s. Morphometric analysis of F1 generation revealed marked reductions in body width and pharynx length, while no statistically significant difference was observed in body length compared to control groups (Fig 8F-8H).

Taken together, our findings demonstrate that insulin signaling plays a key role in iL3 developmental regulation by selectively activating the *Ss*-DAF-2B. The activation of *Ss*-DAF-2B amplifies its transcriptional expression, while exerting no detectable effect on *Ss-daf-2a* transcription. This isoform-specific regulatory pattern indicates that insulin signaling modulates iL3 development via a feedback loop exclusively targeting *Ss-daf-2b* expression. It is a mechanism ensuring precise developmental synchronization with host-derived metabolic cues.

**Fig 9:**
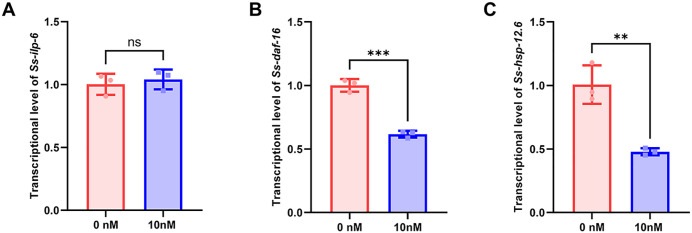
Effects of human insulin on the expression of key insulin signaling genes in *S. stercoralis* iL3s. iL3s were treated with 10 nM human insulin for 12 h, and the mRNA levels of *Ss-daf-16* (A), *Ss-ilp-6* (B) and *Ss-hsp-12.6* (C) were quantified by relative quantitative RT-PCR. Expression levels were normalized to the internal control gene and shown as fold change relative to the control group. Statistical significance was determined using the unpaired two-tailed t-test. Error bars, s.d. ns = not significant, **p < 0.01, ***p < 0.001.

To further investigate whether this subtype-specific activation is accompanied by downstream transcriptional changes in the insulin signaling pathway, we analyzed the expression of key pathway-related genes in *S. stercoralis* iL3s after 12 h of treatment with 10 nM human insulin. We focused on *Ss-*ILP-6, a predicted agonist-type insulin-like peptide, and *Ss*-DAF-16, a core downstream transcription factor. The results showed that the expression level of *Ss-daf-16* was significantly downregulated, while no significant difference was observed in the expression of *Ss-ilp-6* (Fig 9A-9B). As a key effector of the insulin signaling pathway, the downregulation of *Ss-daf-16* is consistent with its functional inhibition during the transition from metabolic arrest to active development. We then examined *Ss-hsp-12.6* (WormBase ID: SSTP_0000790400). *Ce-hsp-12.6* has been validated as a direct target of DAF-16 and is downregulated during dauer exit in *C. elegans* [46]. Similarly, the *A. caninum* ortholog (*Ac-hsp-12.6*) is markedly reduced upon serum stimulation [51]. Consistently, we found that the *S. stercoralis* ortholog *hsp-12.6* was also significantly downregulated in response to insulin treatment (Fig 9C). This result further supports the observed phenotypic shift of iL3s from an arrested to an active state. It indicates that human insulin not only rapidly induces the resumption of feeding behavior but also promotes the developmental process of iL3s by suppressing *Ss-daf-16* transcription. The absence of significant change in *Ss-ilp-6* suggests that under exogenous insulin stimulation, endogenous agonist-type ILPs are not the primary drivers of this transition; instead, host-derived insulin can directly exert this effect. This demonstrates that the parasite can sensitively perceive host signals and regulate its own development accordingly, without relying on the upregulation of endogenous ILPs.

## 4. Discussion

In this study, we first implemented a ligand-dependent OFF-type hammerhead ribozyme switch system in *S. stercoralis* to regulate target gene expression. This system utilizes the low-toxicity theophylline as a modulator to precisely control ribozyme cleavage activity through ligand binding. By applying this system, we achieved dose- and time-dependent suppression of the transcriptional level of the reporter gene *mrfp*. This provides a controllable and efficient platform for gene expression regulation. However, we observed intrinsic structural instability in the ribozyme, which manifested as spontaneous self-cleavage activity. This spontaneous catalytic cleavage significantly reduced baseline transcriptional activity, leading to unintended gene suppression in the absence of the ligand and impairing system controllability. To overcome this challenge, we introduced a non-coding RNA scaffold designed to stabilize the catalytic core of the ribozyme. This RNA scaffold enhanced the conformational stability of the ribozyme’s catalytic active site, effectively minimizing spontaneous cleavage. Through this strategy, we successfully achieved precise regulation of the ribozyme, enabling stable and precise control of target gene expression under the action of the ligand. Besides, a notable limitation of the study is that ribozyme switch system does not yet allow for precise spatial control of endogenous gene downregulation. While spatial regulation of exogenous gene expression can be achieved by driving transcription with tissue-specific promoters, applying the same strategy to endogenous transcripts is more complex. In principle, the ribozyme could be expressed under tissue-specific promoters, which would restrict its activity to defined tissues. However, the targeting efficiency of such tissue-specific ribozymes remains to be further studied. Additionally, their effectiveness would ultimately depend on the distribution and accessibility of the target mRNA within the tissue of interest. This remains an important issue to be addressed in our future work. We also identified several unique advantages of this ribozyme switch system that enhance its utility in studying molecular mechanisms of parasitic nematodes. Small-molecule ligands (e.g., theophylline) can permeate directly through the nematode cuticle without requiring complex delivery vehicles. Furthermore, unlike conventional genetic tools, such as CRISPR-Cas9, this system avoids permanent genomic alterations. By precisely controlling the timing of ligand addition, ribozyme activity can be dynamically regulated, enabling time-specific modulation of target gene expression.

Here, we used this tool to conditionally regulate key molecules in *S. stercoralis*, allowing us to study their role in mediating host-parasite molecular dialogue and parasite development. Parasitic nematodes undergo developmental transitions in close interaction with host’s endocrine and paracrine systems [52–54]. These processes are regulated by evolutionarily conserved hormones, which act as molecular bridges for interactions between the host and the parasite. Parasitic nematodes express receptors, particularly kinase-coupled receptors, that allow them to interact precisely with the host’s endocrine signals. This hormone-mediated cross-species communication between the parasite and the host may be one of the key regulatory mechanisms in parasite development [55–57]. During parasite infection, the insulin receptor (IR) of parasitic nematodes regulates development and metabolism through activation by insulin-related peptides. It has been proposed that, given the widespread conservation of insulin receptor antagonists, no known agonists of DAF-2 have been identified in parasitic nematodes, and host insulins may act as a complementary source of agonists for the insulin receptor [58]. Our study showed the important role of the insulin receptor *Ss*-DAF-2 in the developmental transition of *S. stercoralis*. While previous studies have established that 8-Br-cGMP at 200 μM effectively stimulates approximately 85.1% of iL3s to resume feeding within 24 h supporting its role as a primary stimulus. Our data reveal a crucial kinetic distinction: human insulin induces significant feeding reactivation within just 3 h, whereas 8-Br-cGMP requires at least 12 h to achieve a comparable effect. This rapid response, mediated through the direct activation of *Ss*-DAF-2 by human insulin, highlights its function as an early signal that enables iL3s to quickly recognize the host environment and initiate developmental programs within the first hours of infection, thereby facilitating immune evasion and successful establishment of infection. At the molecular level, we confirmed that human insulin treatment rapidly downregulates the key transcription factor *Ss-daf-16*, further supporting the functional activation of the insulin signaling pathway. The specificity of this interaction is underscored by the inability of non-host mammalian insulins (e.g., porcine or bovine), despite their structural similarity, to activate *Ss*-DAF-2, emphasizing the high selectivity of hormone recognition and suggesting that even minor structural differences can disrupt cross-species signaling. Our study provides the first evidence that host insulin plays a role in breaking developmental arrest in parasitic nematode. *Ss*-DAF-2 acts as a molecular sensor, enabling the parasite to decode host endocrine cues and finely regulate its development. This finding is consistent with the unique architecture of developmental arrest pathway in *S. stercoralis*, where IIS acts at the most downstream position. Both cGMP and DAF-12 NHR signaling operate upstream of IIS to regulate iL3 activation [45]. This evolution underscores the unique and important role of the IIS pathway as the final integrator of developmental cues in host-parasite interactions in parasitic nematodes like *S. stercoralis*. Previous studies have shown that the *Ss-daf-2* gene in *S. stercoralis* is compact (~4.5 kb) and produces two key receptor isoforms via alternative splicing. These two transcript isoforms differ by only a single exon. This mechanism may enable the nematode to discriminate between host and environmental signals during development [48]. In contrast, the *daf-2* gene in the obligate parasite *N. americanus* is simplified to a single receptor, indicating a loss of reliance on complex environmental cues and a focus on parasitic development within the host. This progressive simplification from multiple isoforms to a single form reveals a molecular evolutionary trend of structural and functional focusing in signaling pathways during the transition to parasitism, likely an adaptation for efficiency in the stable host environment. Here, our research focuses on investigating whether the *S. stercoralis* insulin receptor isoforms *Ss*-DAF-2A and *Ss*-DAF-2B exhibit identical binding modes for host-derived ligands. We propose the first 3D molecular structural model of human insulin-*Ss*-DAF-2B coupling. Molecular docking simulations revealed that human insulin selectively binds to *Ss*-DAF-2B but not *Ss*-DAF-2A. This interaction was further validated using a yeast two-hybrid system. Our findings reveal a striking isoform-specific interaction between human insulin and *Ss*-DAF-2B. The selective binding of human insulin to *Ss*-DAF-2B, but not *Ss*-DAF-2A, suggests functional divergence between isoforms that may reflect the adaptive evolutionary refinement of *S. stercoralis* at the host-parasite signaling interface. *Ss*-DAF-2A shows a broader binding capacity, enabling interactions with various endogenous insulin-like peptides in *S. stercoralis*. This suggests functional complementarity between the two isoforms: *Ss*-DAF-2A likely serves to perceive and integrate endogenous parasitic signals for maintaining basal metabolism and physiological activities, whereas *Ss*-DAF-2B has evolved as a host-signal-specific receptor, specialized in recognizing and responding to host insulin molecules to trigger the developmental transition from larval arrest to parasitic adulthood at critical life-cycle checkpoints. This isoform specialization may reflect a strategy: parasites have evolved a receptor system that can exploit conserved host hormones to synchronize their life cycles with host physiology [59–62]. Specifically, *Ss*-DAF-2B may have evolved to adapt to structural changes in host insulin, enabling it to recognize and respond to insulin derived from the host. This mechanism not only shows how parasites use host hormones to regulate their life cycle but also reveals the highly refined molecular-level interactions between parasites and hosts. The evolutionary significance of this isoform-specific interaction needs further research. Is this isoform-specific insulin sensing unique to parasitic nematodes, or does it represent a conserved mechanism in both free-living and parasitic nematodes? Although our work establishes its critical role in *S. stercoralis*, more experimental data are needed to verify the universality of this mechanism in other species.

Due to the characteristics of the *Ss-daf-2* isoforms, we designed ribozyme switch to specifically target the unique exon sequence of *Ss-daf-2b*. This approach enabled highly selective, timed reduction of *Ss-daf-2b* transcription. The results support the necessity of *Ss*-DAF-2B in human insulin-mediated feeding reactivation of iL3. It directly links receptor isoform abundance to parasitic developmental plasticity. This observation parallels studies in *C. elegans*, where IIS pathway modulation alters dauer formation and lifespan [63–66]. Here, we extend the paradigm to parasitic nutrient-sensing mechanisms, revealing the adaptive role of the insulin receptor in host-parasite interactions. Researchers have demonstrated that insulin receptor mRNA abundance is sensitive to both intrinsic and extrinsic disturbances in a cell- and environment-dependent manner [67–70]. The sensitivity of insulin receptor mRNA to hormonal stimuli is likely critical for synchronizing parasitic development with dynamic host metabolic states. Therefore, we analyzed the transcriptional profiling of DAF-2 isoforms. The results showed that host insulin specifically upregulated total *Ss-daf-2* expression, primarily through increased *Ss-daf-2b* transcript levels. This result not only validates the crucial role of insulin signaling in receptor regulation but also underscores the important position of *Ss-daf-2b* in parasite development. In addition, we observed significant reductions in body width and pharynx length in larvae with reduced *Ss-daf-2b* expression. Despite this, these larvae still successfully developed into iL3. *Ss-daf-2b* has some morphological impact on the parasite during development, it is not an essential factor for entering the formation of the iL3 stage. These findings further suggest that *Ss-daf-2b* is a developmental regulator that enables rapid detection of host-derived insulin signals to precisely modulate developmental progression. However, it is not necessary for the entry into the iL3 stage. The preferential upregulation of *Ss-daf-2b* transcripts by host insulin may reflect a feedforward loop that amplifies insulin signaling during parasite invasion into host. Despite fluctuations in host hormone levels, this mechanism ensures that the parasite’s developmental process is not severely disrupted by changes in the host environment.

In conclusion, our findings demonstrate that the ribozyme switch system is functionally operational in parasitic nematodes, enabling targeted regulation of genes of interest. It opens up a pathway for gene function studies in non-model nematodes. Furthermore, ribozyme-mediated RNA targeting enables isoform-specific gene regulation, providing a novel methodology for studying functional differences between isoforms in parasitic nematodes. Employing this system, we revealed that human insulin specifically binds to *Ss*-DAF-2B, inducing a significant upregulation of its transcription. This research establishes the applicability of aptazyme-based tools in *S. stercoralis*. In addition, we elucidated how parasitic nematodes respond to host endocrine signals through receptor isoform specialization, offering both mechanistic insights into host-parasite communication and a roadmap for targeted antihelminthic strategies.

## Supporting information

S1 Table Sequences of primers used for relative quantitative RT-PCR. (DOCX)

S2 Table Aptamer ribozyme switch sequences. (DOCX)

S1 Fig Diagrams of constructs used to transform *Strongyloides stercoralis.* (A) Diagrams of constructs used to transform *S. stercoralis*. The tissue-specific ribozyme targeting plasmid includes a tissue-selective promoter driving expression of the ribozyme. The dual fluorescent reporter system includes *mrfp*, serving as a cleavage-dependent reporter, and *gfp* constitutively expressed under the *Ss-act-2* promoter, acting as an internal control for normalization. (B) Core components of the theophylline-dependent ribozyme switch. Blue: aptamer domain; black: catalytic hammerhead ribozyme core enabling site-specific RNA cleavage; purple: the unique exon sequence of target gene complementary to target hybridization arms; red: flexible linker nucleotides connecting the aptamer and catalytic core; control: ribozyme with non-cleaving mutant.
(TIF)

S2 Fig High concentration of theophylline has an impact on the reproductive capacity of free-living adults and the development of larvae in *Strongyloides stercoralis.* (A) Reproductive capacity of free-living female adults under varying theophylline concentrations. Adult females were paired with males at a ratio of 1:2. Five worms per group were used and the experiment was conducted with five biological replicates. Error bars, s.d. *p < 0.05. Developmental progression of larvae exposed to theophylline was analyzed. (B-F) Body length alterations were quantified at (B) 24 h (n = 31), (C) 48 h (n = 33), and (E) 72 h (n = 35) post-exposure. Additionally, (D) pharynx-to-body length was determined at 48 h (n = 51), while (F) body width was measured following 72 h of drug exposure (n = 36). Statistical significance was determined using the one-way ANOVA and values bearing different superscript letters (a, b and c) differ significantly from one another. Error bars, s.d. *p < 0.05, **p < 0.01, ***p < 0.001, ****p < 0.0001.
(TIF)

S3 Fig The regulatory efficacy of the ribozyme switch on target genes is validated in *Strongyloides stercoralis.* (A) Ribozyme activity was assessed following 24 h exposure to varying theophylline concentrations (0–2 mM). (B) Regulatory capability was evaluated at 1 mM theophylline concentration across multiple time points (0–48 h). Fluorescence intensity was quantified in confocal microscopy images and the results were normalized to untreated controls. Five worms per group were used and experiments were carried out in biological triplicates. Statistical significance was determined using the one-way ANOVA. Error bars, s.d. **p < 0.01, ***p < 0.001, ****p < 0.0001.
(TIF)

S4 Fig Sequence alignment of the ligand-binding domain (LBD) of *Strongyloides stercoralis* with the LBD from other nematode species. Insulin-like receptor ligand-binding domain protein sequences were aligned using Clustal W and visualized as a similarity heatmap generated with TBtools. Color gradient reflects percentage identity (0–100%, scale bar). GenBank accession numbers: *Strongyloides stercoralis* (AGC25444.1), *Haemonchus contortus* (AID54910.1), *Caenorhabditis elegans* (NP_497650.4), *Parastrongyloides trichosuri* (ADN44512.1). Wormbase ID: *Strongyloides ratti* (SRAE_1000288200b.1), *Necator americanus* (Necator_chrIII.pre1.g9420.t1), *Ancylostoma ceylanicum* (Acey_s0036.v2.g328.t1), *Rhabditophanes sp.* (RSKR_0000957200.1).
(TIF)

S5 Fig Yeast two-hybrid assays for detecting the binding interactions between *Strongyloides stercoralis* insulin peptides and *Ss*-DAF-2 receptor isoforms. Translational fusions were generated for the Gal4 activation domain (Gal4-AD) and the endogenous insulin peptides (ILP-1–7) in *S. stercoralis*. The Gal4 DNA binding domain (Gal4-BD) was fused to the insulin receptor *Ss*-DAF-2A and *Ss*-DAF-2B, respectively. Yeast strains were double transformed with the plasmid constructs and growth under different stringency conditions was detected.
(TIF)

S1 Data All raw data accompanying the manuscript. (XLSX)

## References

[1] Santarém VA, Doline FR, Dos Santos JHF, Ferreira IB, Gomes BB, Meisel DMC, et al. Seroprevalence and associated risk factors of strongyloidiasis in indigenous communities and healthcare professionals from Brazil. PLoS Negl Trop Dis. 2023;17(4):e0011283. doi: 10.1371/journal.pntd.0011283 37104537 PMC10168564

[2] Mutombo PN, Man NWY, Nejsum P, Ricketson R, Gordon CA, Robertson G, et al. Diagnosis and drug resistance of human soil-transmitted helminth infections: A public health perspective. Adv Parasitol. 2019;104:247–326. doi: 10.1016/bs.apar.2019.02.004 31030770

[3] Dey AR, Begum N, Anisuzzaman, Alim MA, Alam MZ. Multiple anthelmintic resistance in gastrointestinal nematodes of small ruminants in Bangladesh. Parasitol Int. 2020;77:102105. doi: 10.1016/j.parint.2020.102105 32179135

[4] Njom VS, Winks T, Diallo O, Lowe A, Behnke J, Dickman MJ, et al. The effects of plant cysteine proteinases on the nematode cuticle. Parasit Vectors. 2021;14(1):302. doi: 10.1186/s13071-021-04800-8 34090505 PMC8180098

[5] Bull K, Glover MJ, Rose Vineer H, Morgan ER. Increasing resistance to multiple anthelmintic classes in gastrointestinal nematodes on sheep farms in southwest England. Vet Rec. 2022;190(11):e1531. doi: 10.1002/vetr.1531 35338780 PMC9310741

[6] Rehm C, Klauser B, Finke M, Hartig JS. Engineering Aptazyme Switches for Conditional Gene Expression in Mammalian Cells Utilizing an In Vivo Screening Approach. Methods Mol Biol. 2021;2323:199–212. doi: 10.1007/978-1-0716-1499-0_14 34086282

[7] Win MN, Smolke CD. A modular and extensible RNA-based gene-regulatory platform for engineering cellular function. Proc Natl Acad Sci U S A. 2007;104(36):14283–8. doi: 10.1073/pnas.0703961104 17709748 PMC1964840

[8] Soukup GA, Breaker RR. Design of allosteric hammerhead ribozymes activated by ligand-induced structure stabilization. Structure. 1999;7(7):783–91. doi: 10.1016/s0969-2126(99)80102-6 10425680

[9] Soukup GA, Breaker RR. Nucleic acid molecular switches. Trends Biotechnol. 1999;17(12):469–76. doi: 10.1016/s0167-7799(99)01383-9 10557159

[10] Marshall KA, Ellington AD. Training ribozymes to switch. Nat Struct Biol. 1999;6(11):992–4. doi: 10.1038/14872 10542083

[11] Wurmthaler LA, Sack M, Gense K, Hartig JS, Gamerdinger M. A tetracycline-dependent ribozyme switch allows conditional induction of gene expression in Caenorhabditis elegans. Nat Commun. 2019;10(1):491. doi: 10.1038/s41467-019-08412-w 30700719 PMC6353947

[12] Ausländer S, Ketzer P, Hartig JS. A ligand-dependent hammerhead ribozyme switch for controlling mammalian gene expression. Mol Biosyst. 2010;6(5):807–14. doi: 10.1039/b923076a 20567766

[13] Ketzer P, Kaufmann JK, Engelhardt S, Bossow S, von Kalle C, Hartig JS, et al. Artificial riboswitches for gene expression and replication control of DNA and RNA viruses. Proc Natl Acad Sci U S A. 2014;111(5):E554-62. doi: 10.1073/pnas.1318563111 24449891 PMC3918795

[14] Salvail H, Breaker RR. Riboswitches. Curr Biol. 2023;33(9):R343–8. 10.1016/j.cub.2023.03.069 PMC11207198 37160088

[15] Minematsu T, Mimori T, Tanaka M, Tada I. The effect of fatty acids on the developmental direction of Strongyloides ratti first-stage larvae. J Helminthol. 1989;63(2):102–6. doi: 10.1017/s0022149x00008841 2738378

[16] Stasiuk SJ, Scott MJ, Grant WN. Developmental plasticity and the evolution of parasitism in an unusual nematode, Parastrongyloides trichosuri. Evodevo. 2012;3(1):1. doi: 10.1186/2041-9139-3-1 22214222 PMC3293006

[17] Lok JB. Transgenesis in parasitic nematodes: building a better array. Trends Parasitol. 2009;25(8):345–7. doi: 10.1016/j.pt.2009.05.002 19617000 PMC3066082

[18] Lok JB. Nucleic acid transfection and transgenesis in parasitic nematodes. Parasitology. 2012;139(5):574–88. doi: 10.1017/S0031182011001387 21880161 PMC3319118

[19] Lok JB, Shao H, Massey HC, Li X. Transgenesis in Strongyloides and related parasitic nematodes: historical perspectives, current functional genomic applications and progress towards gene disruption and editing. Parasitology. 2017;144(3):327–42. doi: 10.1017/S0031182016000391 27000743 PMC5364836

[20] Schad GA, Hellman ME, Muncey DW. Strongyloides stercoralis: hyperinfection in immunosuppressed dogs. Exp Parasitol. 1984;57(3):287–96. doi: 10.1016/0014-4894(84)90103-6 6723900

[21] Junio AB, Li X, Massey HC Jr, Nolan TJ, Todd Lamitina S, Sundaram MV, et al. Strongyloides stercoralis: cell- and tissue-specific transgene expression and co-transformation with vector constructs incorporating a common multifunctional 3’ UTR. Exp Parasitol. 2008;118(2):253–65. doi: 10.1016/j.exppara.2007.08.018 17945217 PMC2259275

[22] Massey HC Jr, Ball CC, Lok JB. PCR amplification of putative gpa-2 and gpa-3 orthologs from the (A+T)-rich genome of Strongyloides stercoralis. Int J Parasitol. 2001;31(4):377–83. doi: 10.1016/s0020-7519(01)00117-5 11306115

[23] Lok JB. Strongyloides stercoralis: a model for translational research on parasitic nematode biology. WormBook. 2007;:1–18. doi: 10.1895/wormbook.1.134.1 18050500 PMC3092380

[24] Ly K, Reid SJ, Snell RG. Rapid RNA analysis of individual Caenorhabditis elegans. MethodsX. 2015;2:59–63. doi: 10.1016/j.mex.2015.02.002 26150972 PMC4487333

[25] Bargmann CI, Hartwieg E, Horvitz HR. Odorant-selective genes and neurons mediate olfaction in C. elegans. Cell. 1993;74(3):515–27. doi: 10.1016/0092-8674(93)80053-h 8348618

[26] Castelletto ML, Gang SS, Okubo RP, Tselikova AA, Nolan TJ, Platzer EG, et al. Diverse host-seeking behaviors of skin-penetrating nematodes. PLoS Pathog. 2014;10(8):e1004305. doi: 10.1371/journal.ppat.1004305 25121736 PMC4133384

[27] Moerman DG, Benian GM, Barstead RJ, Schriefer LA, Waterston RH. Identification and intracellular localization of the unc-22 gene product of Caenorhabditis elegans. Genes Dev. 1988;2(1):93–105. doi: 10.1101/gad.2.1.93 2833427

[28] Gang SS, Castelletto ML, Bryant AS, Yang E, Mancuso N, Lopez JB, et al. Targeted mutagenesis in a human-parasitic nematode. PLoS Pathog. 2017;13(10):e1006675. doi: 10.1371/journal.ppat.1006675 29016680 PMC5650185

[29] Altschuler EM, Jungreis CA, Sekhar LN, Jannetta PJ, Sheptak PE. Operative treatment of intracranial epidermoid cysts and cholesterol granulomas: report of 21 cases. Neurosurgery. 1990;26(4):606–13; discussion 614. doi: 10.1097/00006123-199004000-00008 2330082

[30] Thompson JD, Higgins DG, Gibson TJ. CLUSTAL W: improving the sensitivity of progressive multiple sequence alignment through sequence weighting, position-specific gap penalties and weight matrix choice. Nucleic Acids Res. 1994;22(22):4673–80. doi: 10.1093/nar/22.22.4673 7984417 PMC308517

[31] Tamura K, Peterson D, Peterson N, Stecher G, Nei M, Kumar S. MEGA5: molecular evolutionary genetics analysis using maximum likelihood, evolutionary distance, and maximum parsimony methods. Mol Biol Evol. 2011;28(10):2731–9. doi: 10.1093/molbev/msr121 21546353 PMC3203626

[32] Chen C, Chen H, Zhang Y, Thomas HR, Frank MH, He Y, et al. TBtools: An Integrative Toolkit Developed for Interactive Analyses of Big Biological Data. Mol Plant. 2020;13(8):1194–202. doi: 10.1016/j.molp.2020.06.009 32585190

[33] Jumper J, Evans R, Pritzel A, Green T, Figurnov M, Ronneberger O, et al. Highly accurate protein structure prediction with AlphaFold. Nature. 2021;596(7873):583–9. doi: 10.1038/s41586-021-03819-2 34265844 PMC8371605

[34] Yan Y, Tao H, He J, Huang S-Y. The HDOCK server for integrated protein-protein docking. Nat Protoc. 2020;15(5):1829–52. doi: 10.1038/s41596-020-0312-x 32269383

[35] Xue LC, Rodrigues JP, Kastritis PL, Bonvin AM, Vangone A. PRODIGY: a web server for predicting the binding affinity of protein-protein complexes. Bioinformatics. 2016;32(23):3676–8. doi: 10.1093/bioinformatics/btw514 27503228

[36] Nolan TJ, Megyeri Z, Bhopale VM, Schad GA. Strongyloides stercoralis: the first rodent model for uncomplicated and hyperinfective strongyloidiasis, the Mongolian gerbil (Meriones unguiculatus). J Infect Dis. 1993;168(6):1479–84. doi: 10.1093/infdis/168.6.1479 8245532

[37] Ashton FT, Zhu X, Boston R, Lok JB, Schad GA. Strongyloides stercoralis: Amphidial neuron pair ASJ triggers significant resumption of development by infective larvae under host-mimicking in vitro conditions. Exp Parasitol. 2007;115(1):92–7. doi: 10.1016/j.exppara.2006.08.010 17067579 PMC3091007

[38] Wang Z, Zhou XE, Motola DL, Gao X, Suino-Powell K, Conneely A, et al. Identification of the nuclear receptor DAF-12 as a therapeutic target in parasitic nematodes. Proc Natl Acad Sci U S A. 2009;106(23):9138–43. doi: 10.1073/pnas.0904064106 19497877 PMC2695123

[39] Stoltzfus JD, Massey HC Jr, Nolan TJ, Griffith SD, Lok JB. Strongyloides stercoralis age-1: a potential regulator of infective larval development in a parasitic nematode. PLoS One. 2012;7(6):e38587. doi: 10.1371/journal.pone.0038587 22701676 PMC3368883

[40] Ikawa Y, Matsumoto J, Horie S, Inoue T. Redesign of an artificial ligase ribozyme based on the analysis of its structural elements. RNA Biol. 2005;2(4):137–42. doi: 10.4161/rna.2.4.2302 17114929

[41] Porta H, Lizardi PM. An allosteric hammerhead ribozyme. Biotechnology (N Y). 1995;13(2):161–4. doi: 10.1038/nbt0295-161 9634757

[42] Ma L, Huang Z, Liu J. Selection of a self-cleaving ribozyme activated in a chemically and thermally denaturing environment. Chem Commun (Camb). 2021;57(62):7641–4. doi: 10.1039/d1cc03102c 34250983

[43] Huang X, Zhao Y, Pu Q, Liu G, Peng Y, Wang F, et al. Intracellular selection of trans-cleaving hammerhead ribozymes. Nucleic Acids Res. 2019;47(5):2514–22. doi: 10.1093/nar/gkz018 30649474 PMC6412130

[44] Pu Q, Zhou S, Huang X, Yuan Y, Du F, Dong J, et al. Intracellular Selection of Theophylline-Sensitive Hammerhead Aptazyme. Mol Ther Nucleic Acids. 2020;20:400–8. doi: 10.1016/j.omtn.2020.03.001 32244167 PMC7118274

[45] Stoltzfus JD, Bart SM, Lok JB. cGMP and NHR signaling co-regulate expression of insulin-like peptides and developmental activation of infective larvae in Strongyloides stercoralis. PLoS Pathog. 2014;10(7):e1004235. doi: 10.1371/journal.ppat.1004235 25010340 PMC4092141

[46] Datu BJD, Gasser RB, Nagaraj SH, Ong EK, O’Donoghue P, McInnes R, et al. Transcriptional changes in the hookworm, Ancylostoma caninum, during the transition from a free-living to a parasitic larva. PLoS Negl Trop Dis. 2008;2(1):e130. doi: 10.1371/journal.pntd.0000130 18235850 PMC2217673

[47] Dulovic A, Renahan T, Röseler W, Rödelsperger C, Rose AM, Streit A. Rhabditophanes diutinus a parthenogenetic clade IV nematode with dauer larvae. PLoS Pathog. 2020;16(12):e1009113. doi: 10.1371/journal.ppat.1009113 33270811 PMC7738172

[48] Massey HC Jr, Ranjit N, Stoltzfus JD, Lok JB. Strongyloides stercoralis daf-2 encodes a divergent ortholog of Caenorhabditis elegans DAF-2. Int J Parasitol. 2013;43(7):515–20. doi: 10.1016/j.ijpara.2013.01.008 23500073 PMC3648630

[49] Puig O, Marr MT, Ruhf ML, Tjian R. Control of cell number by Drosophila FOXO: downstream and feedback regulation of the insulin receptor pathway. Genes Dev. 2003;17(16):2006–20. doi: 10.1101/gad.1098703 12893776 PMC196255

[50] Puig O, Tjian R. Transcriptional feedback control of insulin receptor by dFOXO/FOXO1. Genes Dev. 2005;19(20):2435–46. doi: 10.1101/gad.1340505 16230533 PMC1257398

[51] Murphy CT, McCarroll SA, Bargmann CI, Fraser A, Kamath RS, Ahringer J, et al. Genes that act downstream of DAF-16 to influence the lifespan of Caenorhabditis elegans. Nature. 2003;424(6946):277–83. doi: 10.1038/nature01789 12845331

[52] Hemphill A, Stettler M, Walker M, Siles-Lucas M, Fink R, Gottstein B. Culture of Echinococcus multilocularis metacestodes: an alternative to animal use. Trends Parasitol. 2002;18(10):445–51. doi: 10.1016/s1471-4922(02)02346-2 12377595

[53] Brehm K. Echinococcus multilocularis as an experimental model in stem cell research and molecular host-parasite interaction. Parasitology. 2010;137(3):537–55. doi: 10.1017/S0031182009991727 19961652

[54] Beall MJ, Pearce EJ. Transforming growth factor-beta and insulin-like signalling pathways in parasitic helminths. Int J Parasitol. 2002;32(4):399–404. doi: 10.1016/s0020-7519(01)00348-4 11849636

[55] Brehm K, Spiliotis M, Zavala-Góngora R, Konrad C, Frosch M. The molecular mechanisms of larval cestode development: first steps into an unknown world. Parasitol Int. 2006;55 Suppl:S15-21. doi: 10.1016/j.parint.2005.11.003 16343987

[56] Gelmedin V, Spiliotis M, Brehm K. Molecular characterisation of MEK1/2- and MKK3/6-like mitogen-activated protein kinase kinases (MAPKK) from the fox tapeworm Echinococcus multilocularis. Int J Parasitol. 2010;40(5):555–67. doi: 10.1016/j.ijpara.2009.10.009 19887070

[57] Brehm K. The role of evolutionarily conserved signalling systems in Echinococcus multilocularis development and host-parasite interaction. Med Microbiol Immunol. 2010;199(3):247–59. doi: 10.1007/s00430-010-0154-1 20376483

[58] Khayath N, Vicogne J, Ahier A, BenYounes A, Konrad C, Trolet J, et al. Diversification of the insulin receptor family in the helminth parasite Schistosoma mansoni. FEBS J. 2007;274(3):659–76. doi: 10.1111/j.1742-4658.2006.05610.x 17181541

[59] Ma G, Wang T, Korhonen PK, Hofmann A, Sternberg PW, Young ND, et al. Elucidating the molecular and developmental biology of parasitic nematodes: Moving to a multiomics paradigm. Adv Parasitol. 2020;108:175–229. doi: 10.1016/bs.apar.2019.12.005 32291085

[60] Hawdon JM, Schad GA. Albumin and a dialyzable serum factor stimulate feeding in vitro by third-stage larvae of the canine hookworm Ancylostoma caninum. J Parasitol. 1991;77(4):587–91. doi: 10.2307/3283164 1713962

[61] Long T, Alberich M, André F, Menez C, Prichard RK, Lespine A. The development of the dog heartworm is highly sensitive to sterols which activate the orthologue of the nuclear receptor DAF-12. Sci Rep. 2020;10(1):11207. doi: 10.1038/s41598-020-67466-9 32641726 PMC7343802

[62] Zhi X, Zhou XE, Melcher K, Motola DL, Gelmedin V, Hawdon J, et al. Structural conservation of ligand binding reveals a bile acid-like signaling pathway in nematodes. J Biol Chem. 2012;287(7):4894–903. doi: 10.1074/jbc.M111.315242 22170062 PMC3281614

[63] Antebi A. Genetics of aging in Caenorhabditis elegans. PLoS Genet. 2007;3(9):1565–71. doi: 10.1371/journal.pgen.0030129 17907808 PMC1994694

[64] Barbieri M, Bonafè M, Franceschi C, Paolisso G. Insulin/IGF-I-signaling pathway: an evolutionarily conserved mechanism of longevity from yeast to humans. Am J Physiol Endocrinol Metab. 2003;285(5):E1064-71. doi: 10.1152/ajpendo.00296.2003 14534077

[65] Kenyon C. The plasticity of aging: insights from long-lived mutants. Cell. 2005;120(4):449–60. doi: 10.1016/j.cell.2005.02.002 15734678

[66] Wolff S, Dillin A. The trifecta of aging in Caenorhabditis elegans. Exp Gerontol. 2006;41(10):894–903. doi: 10.1016/j.exger.2006.06.054 16919905

[67] Norgren S, Li LS, Luthman H. Regulation of human insulin receptor RNA splicing in HepG2 cells: effects of glucocorticoid and low glucose concentration. Biochem Biophys Res Commun. 1994;199(1):277–84. doi: 10.1006/bbrc.1994.1225 8123024

[68] Sesti G, Marini MA, Briata P, Tullio AN, Montemurro A, Borboni P, et al. Androgens increase insulin receptor mRNA levels, insulin binding, and insulin responsiveness in HEp-2 larynx carcinoma cells. Mol Cell Endocrinol. 1992;86(1–2):111–8. doi: 10.1016/0303-7207(92)90181-5 1511777

[69] Takano M, Lu Z, Goto T, Fusi L, Higham J, Francis J, et al. Transcriptional cross talk between the forkhead transcription factor forkhead box O1A and the progesterone receptor coordinates cell cycle regulation and differentiation in human endometrial stromal cells. Mol Endocrinol. 2007;21(10):2334–49. doi: 10.1210/me.2007-0058 17609436

[70] Payankaulam S, Raicu A-M, Arnosti DN. Transcriptional Regulation of INSR, the Insulin Receptor Gene. Genes (Basel). 2019;10(12):984. doi: 10.3390/genes10120984 31795422 PMC6947883

