# Supplementary material for: Aptazyme-mediated gene regulation in Strongyloides stercoralis for functional studies of insulin receptor isoform specificity
Source: PLoS Pathog. 2025 Dec 17;21(12):e1013774. doi: 10.1371/journal.ppat.1013774 (PMC12711028; doi:10.1371/journal.ppat.1013774)
Supplement: S1 Table — (DOCX) [file ppat.1013774.s001.docx]

**S1 Table.** **Sequences of primers used for relative quantitative RT-PCR**

| Primer name | Primer sequence (5'-3') |
| --- | --- |
| Ssbeta-actinRTF | AGCTATGTTCCAACCATCATTCTTGG |
| Ssbeta-actinRTR | CTCCGATCCAGACAGAGTATTTTCTCTC |
| GFPRTF | TGCCATGTGTAATCCCAGCAG |
| GFPRTR | AGACACAACATTGAAGATGGAAGCG |
| mRFPRTF | GAAATTGAAGGTGAAGGTGAAGGTAGAC |
| mRFPRTR | GTTGAATCTTGAGTAACTGTTACAACACCAC |
| Ssunc22RTF | CACAATCTGGAAATGTTCGTCGAAG |
| Ssunc22RTR | TGGTGCAGGATCTCCTTCAACTTTAC |
| Ssdaf16RTF | GAAGCTGCTGGATGGAAGAATTCTATTAG |
| Ssdaf16RTR | CCGTAGACCCATAACATTTAAATGTTCAGTC |
| Ssdaf2bRTF | CTGCAATGTCGATCCTAACCAACC |
| Ssdaf2bRTR | GGTGCATCTAATTGTTCTATTAGTCCACC |
| Ssilp6RTF | GCATTGAATTGTTGTACAAAAGGTTGTAACG |
| Ssilp6RTR | TCTTGATCTTCTTGGGGTTCTATTGC |
| Sshsp12.6RTF | ACAAGCTGATGGTGTTGTTAAAGTTTAT |
| Sshsp12.6RTR | CAACATCATTTGGTAATTTGTATGCACGA |
