# Supplementary material for: Aptazyme-mediated gene regulation in Strongyloides stercoralis for functional studies of insulin receptor isoform specificity
Source: PLoS Pathog. 2025 Dec 17;21(12):e1013774. doi: 10.1371/journal.ppat.1013774 (PMC12711028; doi:10.1371/journal.ppat.1013774)
Supplement: S2 Table — (DOCX) [file ppat.1013774.s002.docx]

**S2 Table.** **Aptamer ribozyme switch sequences**

| Name | Primer sequence (5'-3') |
| --- | --- |
| mRFPHHR | GAGCAGCGGCCGTACTTCCACCAACGAATTCACGAACGAATTCCATGGTAATGC  TGATGAGAGGATACCAGCCGAAAGGCCCTTGGCAGCCTCGAAACCACCTTTTG |
| mRFPunHHR | GAGCAGCGGCCGTACTTCCACCAACGAATTCACGAACGAATTCCATGGTAATGCT  GATGAGAGGATACCAGCCGAAAGGCCCTTGGCAGCCTCGAGACCACCTTTTG |
| Ssunc22HHR | GAGCAGCGGCCGTACTTCCACCAACGAATTCACGAACGAATTCCAGATGGTGGGC  TGATGAGAGGATACCAGCCGAAAGGCCCTTGGCAGCCTCGAAACCAAACTAATT |
| Ssdaf16HHR | GAGCAGCGGCCGTACTTCCACCAACGAATTCACGAACGAATTCCAATTTCCCTGCT  GATGAGTATACCAGCCGAAAGGCCCTTGGCAGACGAAACACTTAAAT |
| Ssdaf16U15HHR | CTTCGATGAAGAGATGATGACGAGTCTGACTTGGGGATGTTCTCTTTGCCCAGGTG  GCCTACTCTGTGCTGCGTTAAAAAATTTCCCTGCTGATGAGTATACCAGCCGAAAGG  CCCTTGGCAGACGAAACACTTAAATAAAACTGTGGCACAGTTTAAAGAGCCCTGGT  TGAAGTAATTTCCTAAAGATGACTTAGAGGCATTTGTCTGAGAAGG |
| Ssdaf2bU15HHR | CTTCGATGAAGAGATGATGACGAGTCTGACTTGGGGATGTTCTCTTTGCCCAGGTG  GCCTACTCTGTGCTGCGTTAAAAATTAGGATCCTGATGAGAGGATACCAGCCGAAA  GGCCCTTGGCAGCCTCGAAACATTGCAGAAAACTGTGGCACAGTTTAAAGAGCCC  TGGTTGAAGTAATTTCCTAAAGATGACTTAGAGGCATTTGTCTGAGAAGG |
| Ssdaf2aU15HHR | CTTCGATGAAGAGATGATGACGAGTCTGACTTGGGGATGTTCTCTTTGCCCAGGTG  GCCTACTCTGTGCTGCGTTAAAAATTAATTCTTTGTCTGATGAGAGGATACCAGCCGAAAGGCCCTTGGCAGCCTCGAATCCATACTAAAACTGTGGCACAGTTTAAAGAGCCC  TGGTTGAAGTAATTTCCTAAAGATGACTTAGAGGCATTTGTCTGAGAAGG |

Blue: U15 sequence; red: linker sequence; black: catalytic hammerhead ribozyme sequences
