# Supplementary figures and images for: Aptazyme-mediated gene regulation in Strongyloides stercoralis for functional studies of insulin receptor isoform specificity
Source: PLoS Pathog. 2025 Dec 17;21(12):e1013774. doi: 10.1371/journal.ppat.1013774 (PMC12711028; doi:10.1371/journal.ppat.1013774)

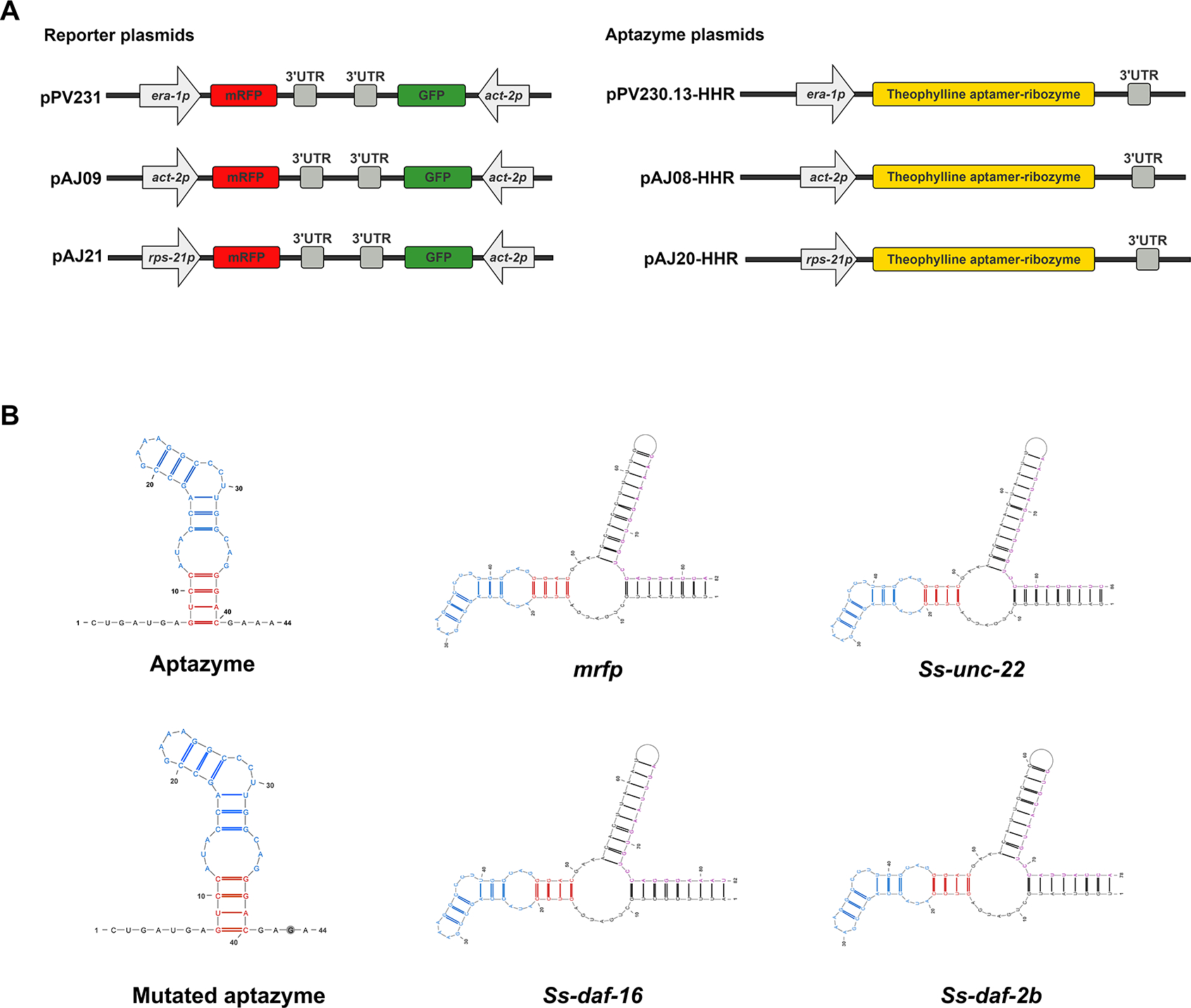

Supplement: S1 Fig — (A) Diagrams of constructs used to transform S. stercoralis. The tissue-specific ribozyme targeting plasmid includes a tissue-selective promoter driving expression of the ribozyme. The dual fluorescent reporter system includes mrfp, serving as a cleavage-dependent reporter, and gfp constitutively expressed under the Ss-act-2 promoter, acting as an internal control for normalization. (B) Core components of the theophylline-dependent ribozyme switch. Blue: aptamer domain; black: catalytic hammerhead ribozyme core enabling site-specific RNA cleavage; purple: the unique exon sequence of target gene complementary to target hybridization arms; red: flexible linker nucleotides connecting the aptamer and catalytic core; control: ribozyme with non-cleaving mutant. (TIF) [file ppat.1013774.s003.tif]

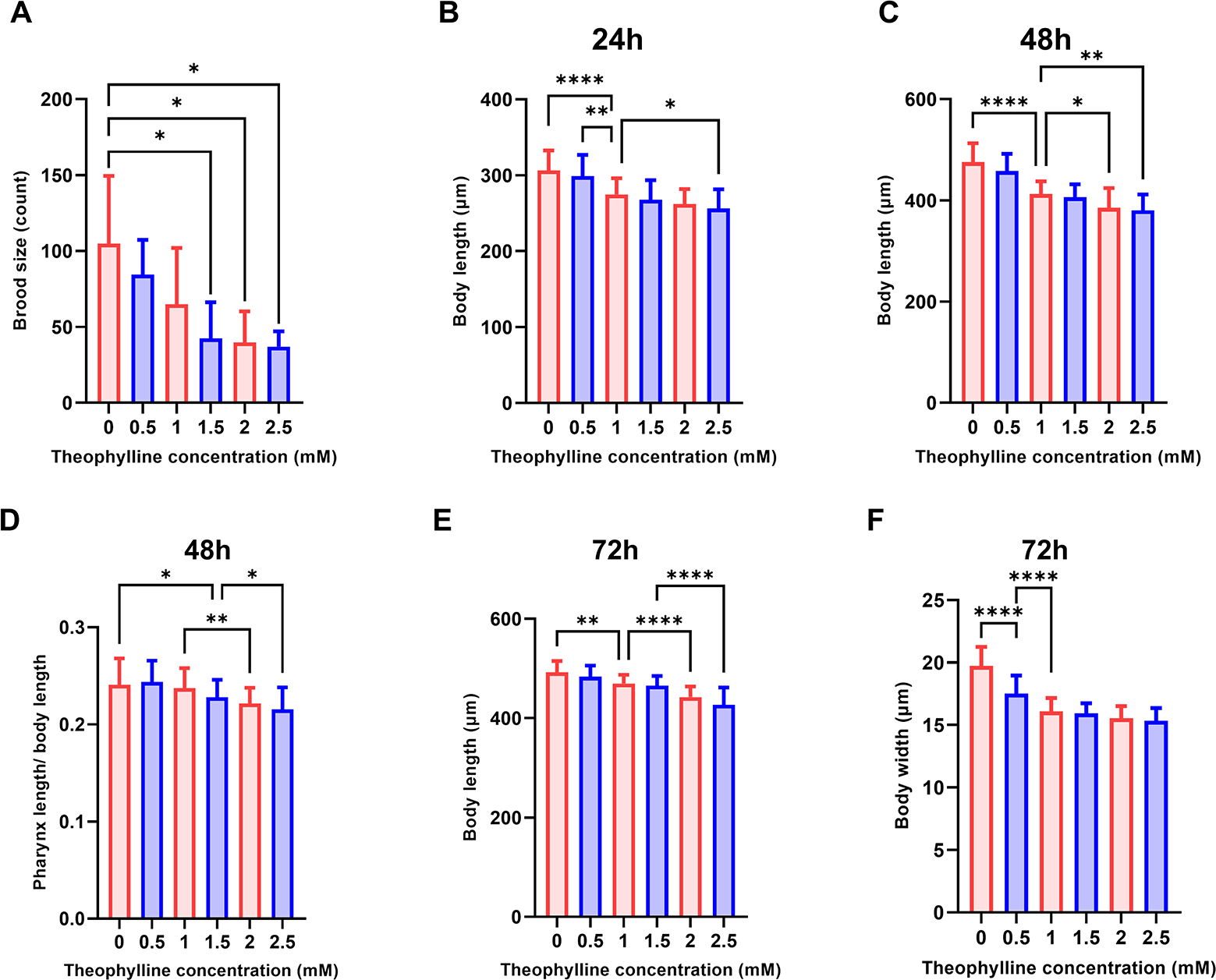

Supplement: S2 Fig — (A) Reproductive capacity of free-living female adults under varying theophylline concentrations. Adult females were paired with males at a ratio of 1:2. Five worms per group were used and the experiment was conducted with five biological replicates. Error bars, s.d. *p < 0.05. Developmental progression of larvae exposed to theophylline was analyzed. (B-F) Body length alterations were quantified at (B) 24 h (n = 31), (C) 48 h (n = 33), and (E) 72 h (n = 35) post-exposure. Additionally, (D) pharynx-to-body length was determined at 48 h (n = 51), while (F) body width was measured following 72 h of drug exposure (n = 36). Statistical significance was determined using the one-way ANOVA and values bearing different superscript letters (a, b and c) differ significantly from one another. Error bars, s.d. *p < 0.05, **p < 0.01, ***p < 0.001, ****p < 0.0001. (TIF) [file ppat.1013774.s004.tif]

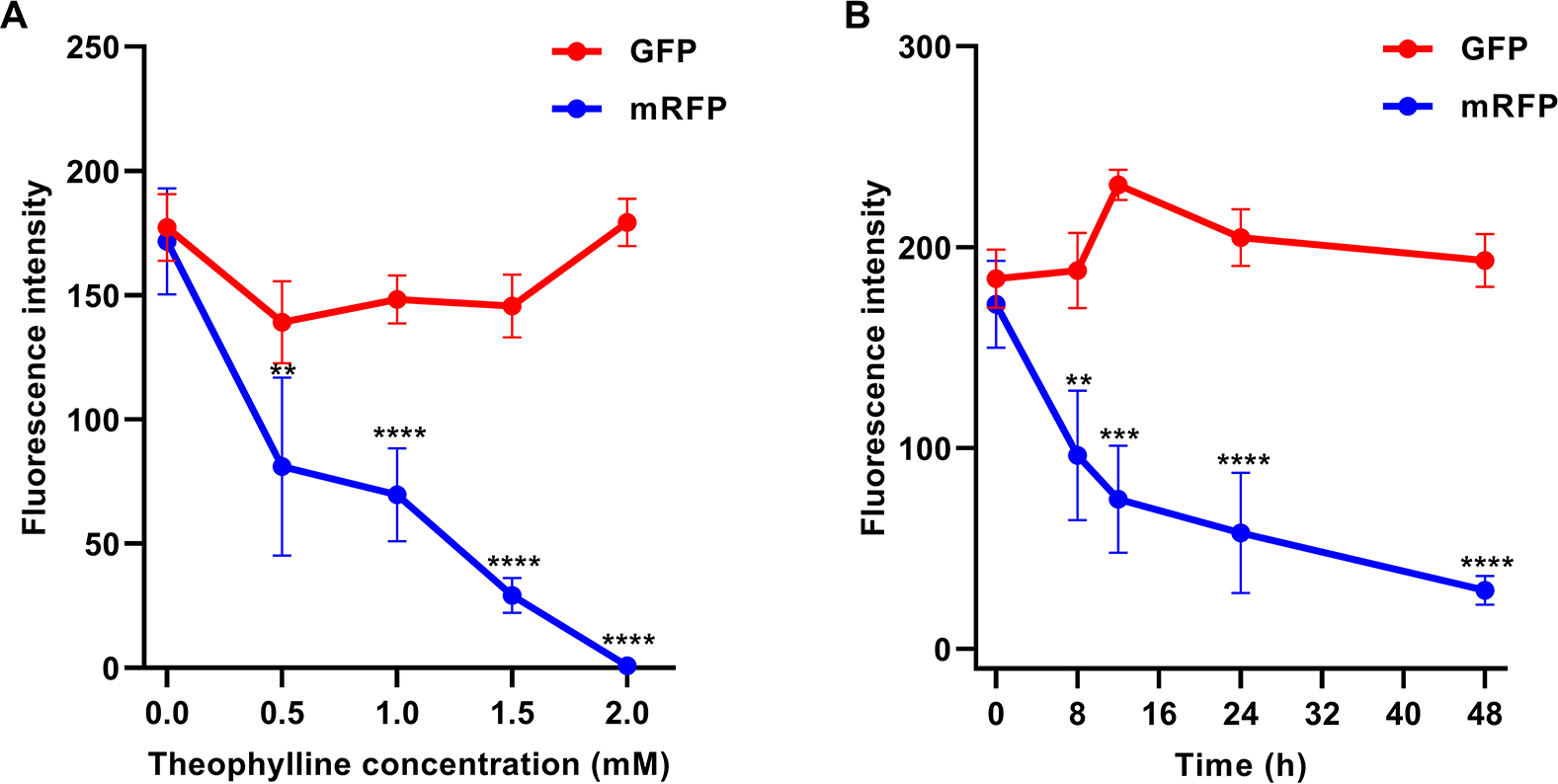

Supplement: S3 Fig — (A) Ribozyme activity was assessed following 24 h exposure to varying theophylline concentrations (0–2 mM). (B) Regulatory capability was evaluated at 1 mM theophylline concentration across multiple time points (0–48 h). Fluorescence intensity was quantified in confocal microscopy images and the results were normalized to untreated controls. Five worms per group were used and experiments were carried out in biological triplicates. Statistical significance was determined using the one-way ANOVA. Error bars, s.d. **p < 0.01, ***p < 0.001, ****p < 0.0001. (TIF) [file ppat.1013774.s005.tif]

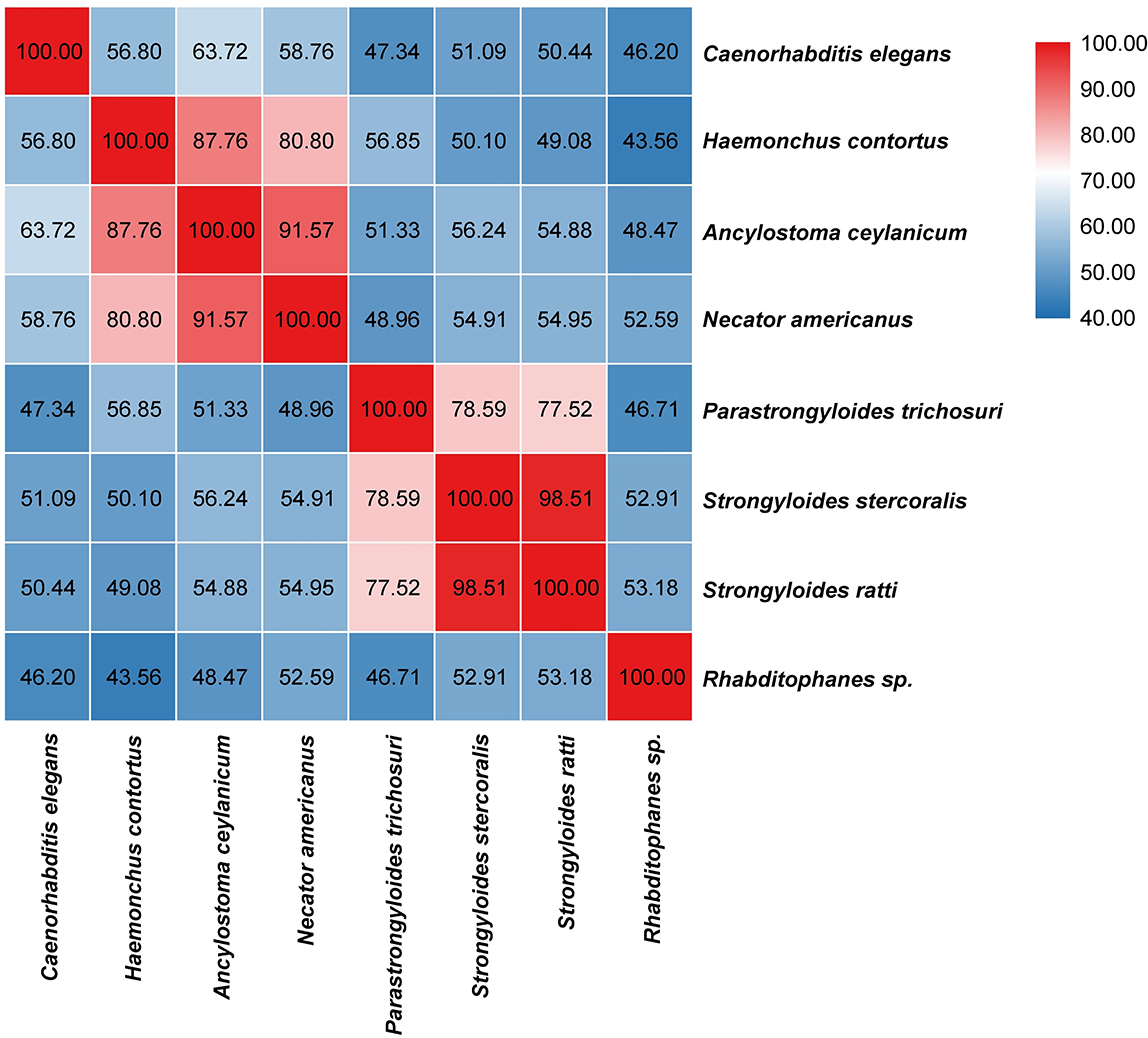

Supplement: S4 Fig — Insulin-like receptor ligand-binding domain protein sequences were aligned using Clustal W and visualized as a similarity heatmap generated with TBtools. Color gradient reflects percentage identity (0–100%, scale bar). GenBank accession numbers: Strongyloides stercoralis (AGC25444.1), Haemonchus contortus (AID54910.1), Caenorhabditis elegans (NP_497650.4), Parastrongyloides trichosuri (ADN44512.1). Wormbase ID: Strongyloides ratti (SRAE_1000288200b.1), Necator americanus (Necator_chrIII.pre1.g9420.t1), Ancylostoma ceylanicum (Acey_s0036.v2.g328.t1), Rhabditophanes sp. (RSKR_0000957200.1). (TIF) [file ppat.1013774.s006.tif]

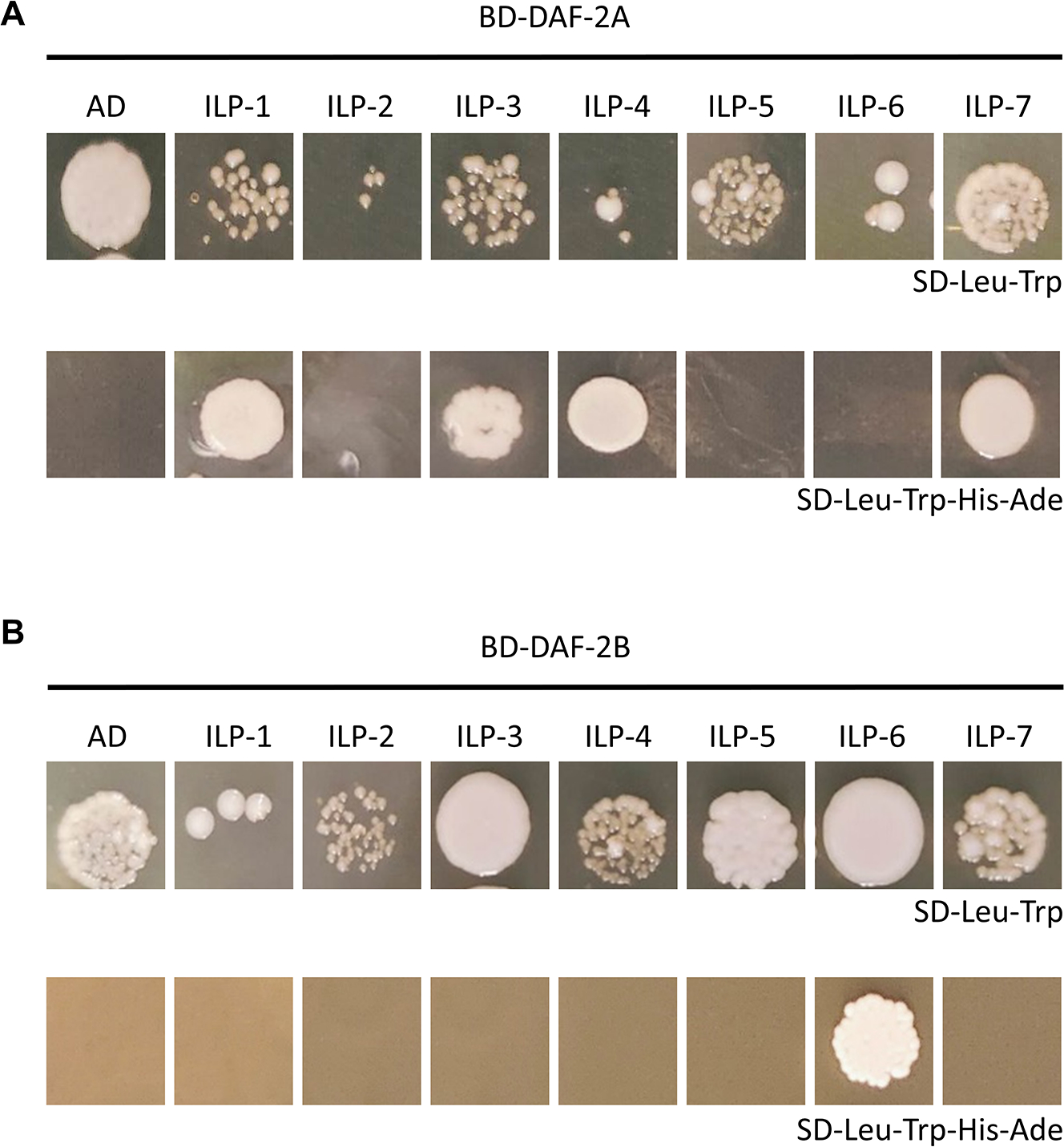

Supplement: S5 Fig — Translational fusions were generated for the Gal4 activation domain (Gal4-AD) and the endogenous insulin peptides (ILP-1–7) in S. stercoralis. The Gal4 DNA binding domain (Gal4-BD) was fused to the insulin receptor Ss-DAF-2A and Ss-DAF-2B, respectively. Yeast strains were double transformed with the plasmid constructs and growth under different stringency conditions was detected. (TIF) [file ppat.1013774.s007.tif]
